# Identification of key genes and diagnostic model associated with circadian rhythms and Parkinson’s disease by bioinformatics analysis

**DOI:** 10.3389/fnagi.2024.1458476

**Published:** 2024-10-16

**Authors:** Jiyuan Zhang, Xiaopeng Ma, Zhiguang Li, Hu Liu, Mei Tian, Ya Wen, Shan Wang, Liang Wang

**Affiliations:** ^1^Department of Neurology, The Second Hospital of Hebei Medical University, Shijiazhuang, China; ^2^School of Basic Medicine, Hebei Medical University, Shijiazhuang, China; ^3^Xingtai Third Hospital, Xingtai, China; ^4^The Key Laboratory of Neurology, Hebei Medical University, Ministry of Education, Shijiazhuang, China; ^5^Neurological Laboratory of Hebei Province, Shijiazhuang, China

**Keywords:** Parkinson’s disease, circadian rhythm, GEO, bioinformatics, biomarkers

## Abstract

**Background:**

Circadian rhythm disruption is typical in Parkinson’s disease (PD) early stage, and it plays an important role in the prognosis of the treatment effect in the advanced stage of PD. There is growing evidence that circadian rhythm genes can influence development of PD. Therefore, this study explored specific regulatory mechanism of circadian genes (C-genes) in PD through bioinformatic approaches.

**Methods:**

Differentially expressed genes (DEGs) between PD and control samples were identified from GSE22491 using differential expression analysis. The key model showing the highest correlation with PD was derived through WGCNA analysis. Then, DEGs, 1,288 C-genes and genes in key module were overlapped for yielding differentially expressed C-genes (DECGs), and they were analyzed for LASSO and SVM-RFE for yielding critical genes. Meanwhile, from GSE22491 and GSE100054, receiver operating characteristic (ROC) was implemented on critical genes to identify biomarkers, and Gene Set Enrichment Analysis (GSEA) was applied for the purpose of exploring pathways involved in biomarkers. Eventually, immune infiltrative analysis was applied for understanding effect of biomarkers on immune microenvironment, and therapeutic drugs which could affect biomarkers expressions were also predicted. Finally, we verified the expression of the genes by q-PCR.

**Results:**

Totally 634 DEGs were yielded between PD and control samples, and MEgreen module had the highest correlation with PD, thus it was defined as key model. Four critical genes (AK3, RTN3, CYP4F2, and LEPR) were identified after performing LASSO and SVM-RFE on 18 DECGs. Through ROC analysis, AK3, RTN3, and LEPR were identified as biomarkers due to their excellent ability to distinguish PD from control samples. Besides, biomarkers were associated with Parkinson’s disease and other functional pathways.

**Conclusion:**

Through bioinformatic analysis, the circadian rhythm related biomarkers were identified (AK3, RTN3 and LEPR) in PD, contributing to studies related to PD treatment.

## 1 Introduction

Parkinson’s disease (PD) is a long-term, progressive neurodegenerative condition that is mainly characterized by motor dysfunction, including bradykinesia, instability in posture, muscle stiffness, and resting tremor (Yang et al., 2020; Aarsland et al., 2021). One distinguishing feature of the illness is the selective loss of dopaminergic neurons in the Substantia Nigra pars compacta (SNpc), which causes motor symptoms (Maiti et al., 2017; Guatteo et al., 2022). Another distinct characteristic of PD is the existence of Lewy bodies, which are cytoplasmic aggregates mostly made of ubiquitin and *α*-synuclein (α-syn), within the remaining neurons (Bérard et al., 2022; Surguchov and Surguchev, 2022; Stoker and Greenland, 2018). The pathophysiology of PD is yet unknown, however, hereditary factors, environmental variables, age, and oxidative stress may all play a role in the degenerative death of dopaminergic neurons (Chang and Chen, 2020; Jankovic and Tan, 2020). Living independently is challenging for PD patients, which places a huge load on individuals and their families. At present, the diagnosis criteria for PD are still dependent only on motor symptoms that appear years after the neurodegenerative process has begun (Tolosa et al., 2021). But the emergence of symptoms linked to dyskinesia has revealed that PD patients are at an advanced clinical stage. Symptoms of mobility abnormalities in PD patients can be avoided as soon as feasible if the neurodegeneration is identified and treated at an early stage.

Although PD is classified as a movement illness, most patients also have a wide range of non-motor symptoms (NMS) (Kulisevsky et al., 2013). NMS often appear in the early stages of PD and often precede motor dysfunction. Nonmotor symptoms such as decreased olfaction, constipation, nocturia, neuropsychiatric symptoms, and sleep difficulties are very prevalent and can occur many years before the clinical diagnosis of PD (Zhang et al., 2016).

The circadian rhythm is a 24-h cycle that governs physiological and behavioral processes (Hood and Amir, 2017). Circadian rhythms control almost all bodily functions and behaviors, including sleep–wake cycles, body temperature, blood pressure, food intake, hormone release, and others (Serin and Acar Tek, 2019). Circadian rhythm disruption is typical in PD early stage. And symptoms associated with circadian rhythms can persist into the later stages of Parkinson’s disease (Hunt et al., 2022). Circadian rhythm disorder can cause rapid eye movement sleep behavior disorder, night insomnia, excessive daytime sleepiness and many other sleep disorders (Gros and Videnovic, 2020). Circadian rhythm disruption also impacts the corresponding fluctuations in blood pressure and heart rate, resulting in hypertension at night and hypotension during the day. Circadian rhythm issues for PD patients may have an impact on depression propensity, treatment responsiveness, body temperature, endocrine abnormalities, and other occurrences (Videnovic and Zee, 2015). Polymorphisms in clock genes have been linked to an increased risk of PD (De Lazzari et al., 2018; Shkodina et al., 2022). It has been postulated that clock gene mutations may contribute to PD pathogenesis by altering circadian regulation of processes such as mitochondrial bioenergetics, autophagy, and neuroendocrine function (Hunt et al., 2022). Many neurological illnesses, including schizophrenia, bipolar disorder, depression, and autism, rely on circadian rhythms (Hunt et al., 2022).

**Figure 1:**
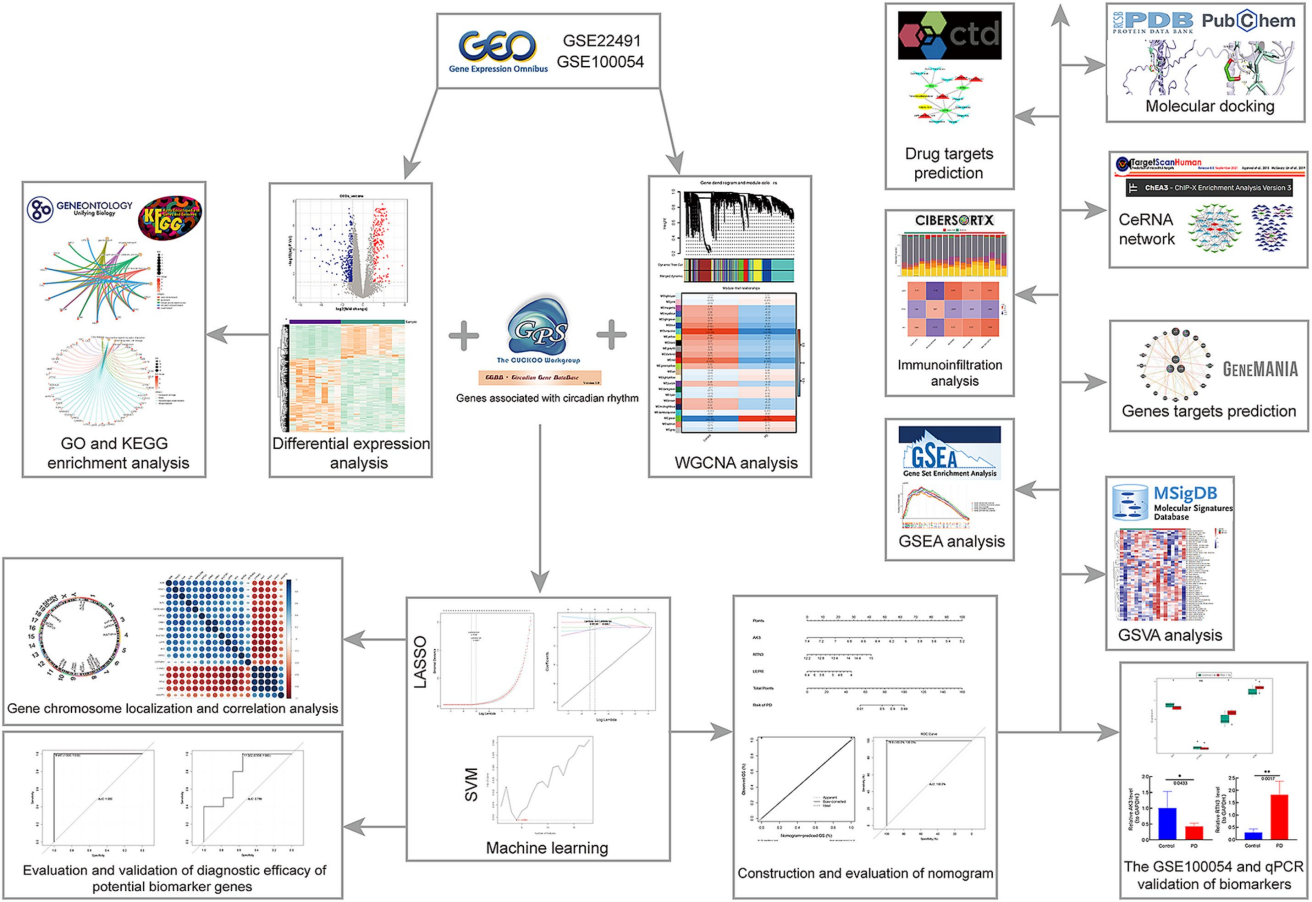
Flow chart of this study.

We hope to investigate the mechanism of circadian rhythm disturbance in Parkinson’s disease patients using high-throughput chip technology, as well as uncover relevant molecular markers that may diagnose and predict PD at an early stage and monitor patients’ circadian rhythm disruption for a long period. We extracted data sets GSE22491 and GSE100054 and 1,288 circadian rhythm genes from a public database. Differential expression analysis, WGCNA, LASSO, SVM-RFE, ROC, etc. were used to find circadian-related PD biomarkers, and genome enrichment analysis (GSEA) was used to investigate the functional pathways connected to PD. Finally, immunoinfiltration analysis was carried out in order to better understand the role of biomarkers in the immune milieu and to suggest therapeutic drugs that may influence biomarker expression. The strategy of this study is shown in Figure 1. It is crucial for the diagnosis and therapy of Parkinson’s disease to understand the mechanism of circadian biomarkers in the illness. The aim of this study was to investigate the regulatory mechanisms of circadian rhythm genes in Parkinson’s disease and to provide new ideas to guide the clinical treatment of Parkinson’s disease patients.

## 2 Materials and methods

### 2.1 Sources of data

Datasets GSE22491 and GSE100054 were acquired via GEO,^1^ in which the GSE22491 included peripheral blood mononuclear cells (PBMC) for 10 Parkinson’s disease (PD) samples and PBMC for 8 control samples (Mutez et al., 2011). GSE100054 contained 8 PD PBMC samples and 8 control PBMC samples (Miki et al., 2018). Then, totally 1,288 circadian genes (C-genes) were extracted via Circadian Gene DataBase (CGDB) database^2^ (Li et al., 2017; Yin X. et al., 2022; Yin Z. et al., 2022).

### 2.2 Differential expression analysis and ingenuity pathway analysis

After acquiring the (GSE22491) dataset, we first applied log_2_(x + 1) normalization to ensure the accuracy and reliability of the subsequent analysis. Subsequently, we performed differential gene analysis using stringent criteria (p.adjust<0.05, |log_2_FC| > 1) (Wang et al., 2021) to accurately screen for genes that were significantly differentially expressed between the disease and control groups. Through this process, genes that were lowly expressed in all samples were virtually automatically excluded, thus eliminating the need for additional screening for lowly expressed genes. Then, for the remaining gene were subjected to GO and KEGG through R package clusterProfiler (version 1.58.0) for finding functional pathways, filter criteria was adj. *p* < 0.05 (Yu et al., 2012). Subsequently, IPA was implemented in GSE22491 for exploring enrichment pathways of DEGs (Krämer et al., 2014). The symbol columns of DEGs and corresponding logFC columns were uploaded to IPA software, and preference was set to 3,000 mb, confirming that analyzed data was consistent with molecular background. Construction of interactions network was set to consider indirect effects, and data filtering threshold of 100–2000 was set to include contents of MyPathways and List created by individual into analysis together, RUN was clicked to start analysis. Z-score > 0 in indicated pathway was activated, and Z-score < 0 indicated inhibited. Role of biomarkers in transmission of signaling pathway corresponding to the highest value of |z-score| was displayed (Wang et al., 2022; Yang et al., 2022). In this study, we chosed thresholds of z-score > 2 or z-score < −2.

### 2.3 WGCNA

In this study, WGCNA analysis was performed on the entire sample of the training set using PD as a trait. WGCNA was applied in GSE22491 via R package WGCNA (version 1.70–3) (Langfelder and Horvath, 2008). GSE22491 samples were clustered for removing outliers, sample clusters were constructed for visualizing results. A soft threshold was chosen on the light of facts with constructed network maximally conformed to scale-free distribution and R2 closed to threshold 0.85. For constructing the co-expression matrix, all detected genes from GSE22491 were clustered into different modules. Therefore, neighboring and similarity were estimated for introducing coefficient of dissimilarity among genes, systematic clustering tree among genes was yielded according to coefficient of dissimilarity. Modules (set MEDissThres = 0.25 to merge similar modules, *p* < 0.05) with minimum number of 100 genes were sifted out for correlations analysis with sample groups (PD and control), key module that was highly correlated with PD was sifted out.

### 2.4 Acquisition of differentially expressed C-genes

The DEGs, C-genes and genes in the key module were overlapped to yield DECGs, and their localization on chromosomes as well as correlations among them were analyzed, GO and KEGG analyses were also applied with threshold *p* < 0.05 to identify relevant functional pathways.

### 2.5 Screening for critical genes via machine learning methods

In order to further screen out biomarkers that could diagnose the PD, we applied machine learning algorithm analyses. First, least absolute shrinkage and selection operator (LASSO) logistic regression algorithm was implemented on DECGs, and gene coefficient and cross-validation error maps were gained. The importance degrees of DECGs were ranked by support vector machine-recursive feature elimination (SVM-RFE) algorithm, and genes with the lowest error rate point were sifted out. Subsequently, critical genes were identified after taking the intersection of genes obtained by LASSO and SVM-RFE, the parameters were set to Lambda.min = 0.0046, AUC > 0.7.

### 2.6 Assessment of biomarkers’ diagnostic value

To evaluate ability of critical genes to distinguish PD from control samples, ROC curves were applied in GSE22491 and GSE100054 via pROC package (version 1.0.3) (Robin et al., 2011). ROC curves are plotted based on different dichotomous thresholds, where the vertical coordinate is the true-positive rate (Sensitivity) and the horizontal coordinate is the false-positive rate (1-Specificity). The closer the AUC (Area Under the Curve) is to 1, the higher the diagnostic accuracy of the model. Our analysis is based on an existing dataset designed to predict the risk of disease prevalence. We used an AUC value of 0.7 as a criterion, and area under the curve (AUC) of critical genes exceed 0.7 both in GSE22491 and GSE100054 were defined as biomarkers.

### 2.7 Construction of the diagnostic model

In GSE22491, the “rms” package (Liu et al., 2021) was used to construct a biomarker-based column plot model for PD risk prediction. Then, using the “pROC” package, we analyzed the ROC analysis (AUC > 0.7) of the column charts and evaluated the performance of the column charts in predicting the risk of PD, and the result was 1, which indicated that the performance was good. In addition, based on the column-line diagram prediction model, we plotted the calibration curves corresponding to the column-line diagrams. The horizontal coordinate of the calibration curve is the predicted probability and the vertical coordinate is the actual probability, and the closer the slope of the calibration curve is to 1, the more accurate the model prediction.

### 2.8 GSEA, gene set variation analysis

To explore the regulatory pathways or biological functions associated with target gene expression, we performed GSEA enrichment analysis. Compared with traditional enrichment analysis, GSEA enrichment analysis does not need to specify explicit differential gene thresholds, and the algorithm analyzes according to the actual overall trend, and does not miss some key information due to unreasonable screening parameters. Therefore, GSEA enrichment analysis can retain the key information without differential screening, and thus find the functional gene sets with insignificant differences but very consistent trends. The purpose of single-gene GSEA analysis is to find the regulatory pathways or biological functions associated with the expression of target genes. In this study, we calculated the correlation coefficients of the expression of all the genes with the target genes as a sorting criterion for the GSEA enrichment analysis.

For purpose of exploring functional pathways involved in biomarkers, GSEA was applied via R package clusterProfiler (version 1.58.0) (Yu et al., 2012) with adj. *p* < 0.05 (background gene set: KEGG: c2.cp.kegg.v2022.1.Hs.entrez.gmt), Individual biomarkers and all genes inside GSE22491 were analyzed for correlation and scored in order of correlation value. Next, expression high/low groups were classified according to biomarkers’ median expression, and significant pathways between these two groups in GSE22491 were displayed in heatmap through R packages clusterProfiler (version 1.58.0) (Yu et al., 2012) and GSVA (version 3. 15.0) (Su et al., 2022) (background gene set: h.all.v7.5.1.symbols.gmt).

### 2.9 Immune infiltrative analysis

To understand role that biomarkers played in immune microenvironment of PD, immune infiltration analysis was carried out in GSE22491. First infiltration abundance of immune cells in GSE22491 samples was estimated via cell type identification by estimating relative subsets of RNA transcripts (CIBERSORT). Spearman correlations among them was analyzed. After that, differences in immune cells’ proportions between PD and control samples from GSE22491 were compared, and sensibly different immune cells were selected for correlation analysis with biomarkers (*p* < 0.05, |r| > 0.3).

### 2.10 Construction of the GeneMANIA, competing endogenous RNA, and mRNA-miRNA-transcription factor networks

For the purpose of understanding the genes associated with biomarkers and functional pathways in which they are involved, GeneMANIA analysis was carried out (Franz et al., 2018). Then, Starbase^3^ (Li et al., 2014) and TargetScan^4^ databases (McGeary et al., 2019; Jia et al., 2022) were utilized to predict miRNAs targeting biomarkers, MiRNAs meeting the criteria with Conserved Sites in TargetScan database and all miRNAs predicted in the Starbase database were selected to determine the intersection and obtain the intersected miRNAs. Following this, lncRNAs targeting the intersected miRNAs were identified via the Starbase database with clipExpNum >15. A ceRNA network was acquire using Cytoscape software based on the above results. Additionally, TFs that regulate biomarkers were predicted from the ChEA3 database^5^ (Keenan et al., 2019), and a mRNA-miRNA-TF network was constructed.

### 2.11 Prediction and molecular docking analysis of potential therapeutic agents

In order to predict the drugs that affect the expression of biomarkers. Therapeutic drugs which targeting multiple biomarkers were predicted using the comparative toxicogenomics database (CTD)^6^ (Davis et al., 2021). Drugs with Interaction Count ≥2 and Organism Count ≥2 were selected to construct a drug-gene network with biomarkers. Subsequently, 3D crystal structures of biomarker encoded proteins were downloaded utilizing Protein Data Bank (PDB)^7^ (Berman et al., 2000), and saved as protein PDB format files. 2D structures of drugs were obtained from the PubChem database^8^ (Kim et al., 2023), and saved in SDF format, OpenBabel software was applied to convert them to PDB format for small molecule ligands. Next, water molecules and original ligands were removed by using PyMOL software, and the proteins and small molecules’ PDB files were converted to pdbqt format utilizing AutoDockTools software. Protein receptors and small molecule ligands were docked by AutoDock using.

### 2.12 External gene set validation

In order to assess biomarkers’ expression levels of between PD and control samples in this study, their expression trends were demonstrated by plotting box plots in GSE22491. Meanwhile, expression levels were further validated in GSE100054.

### 2.13 Patient sample collection and ethical clearance

We collected blood samples from 5 PD patients and 5 controls from the Second Hospital of Hebei Medical University. This study is consistent with the Declaration of Helsinki. The study involving human participants was reviewed and approved by the Ethics Committee of the Second Hospital of Hebei Medical University, and all included patients signed written informed consent for the study.

### 2.14 Quantitative real-time polymerase chain reaction

To demonstrate the expression levels of the biomarkers in GSE22491 and GSE100054. Total RNA from the 10 samples was extracted using the TRIzol reagent (Invitrogen, China) according to the manufacturer’s protocol. Afterwards, RNA concentrations were detected with NanoPhotometer N50. Subsequently, cDNA was generated through reverse transcription using SureScript-First-strand cDNA synthesis kit (Servicebio, China). Finally, the qPCR assay was conducted using CFX Connect Thermal Cycler (Bio-Rad, United States). The relative quantification of mRNAs was calculated using the 2^-ΔΔCT^ method. The sequence fragments of RNAs are shown in Table 1.

**Table 1:** PCR primers.

Gene	Forward primer sequence	Reverse primer sequence
AK3	CCTGATCAGTCAGCAGCCATC	GCCTGTGGAAGTGTCCTTGG
RTN3	AAGGCCATCCATTCAAACCCA	AACAATTGACTTGGTCTGATCTCG
CYP4F2	GGTCATCTCCCGCCATGT	CTGGGTTGTGATGGGTTCCG
LEPR	CCATCTCTGCCTTCGGTCG	TCCAGCAGGCAAAAGGAAGT
GAPDH	CGAAGGTGGAGTCAACGGATTT	ATGGGTGGAATCATATTGGAAC

### 2.15 Statistical analysis

Statistical analysis was carried out through R software (version R-4.2.2).^9^ Differences between samples were analyzed via Wilcox test and *t*-test. *p < 0.05, **p < 0.005, ***p < 0.0005; ****p < 0.00005, represented significant difference. In GSEA, judgment standard was adj. *p* < 0.05. Correlation coefficients and *p*-values were estimated utilizing Spearman correlation analysis. *p* < 0.05 was considered statistically significant.

## 3 Results

### 3.1 Identification of DEGs

**Figure 2:**
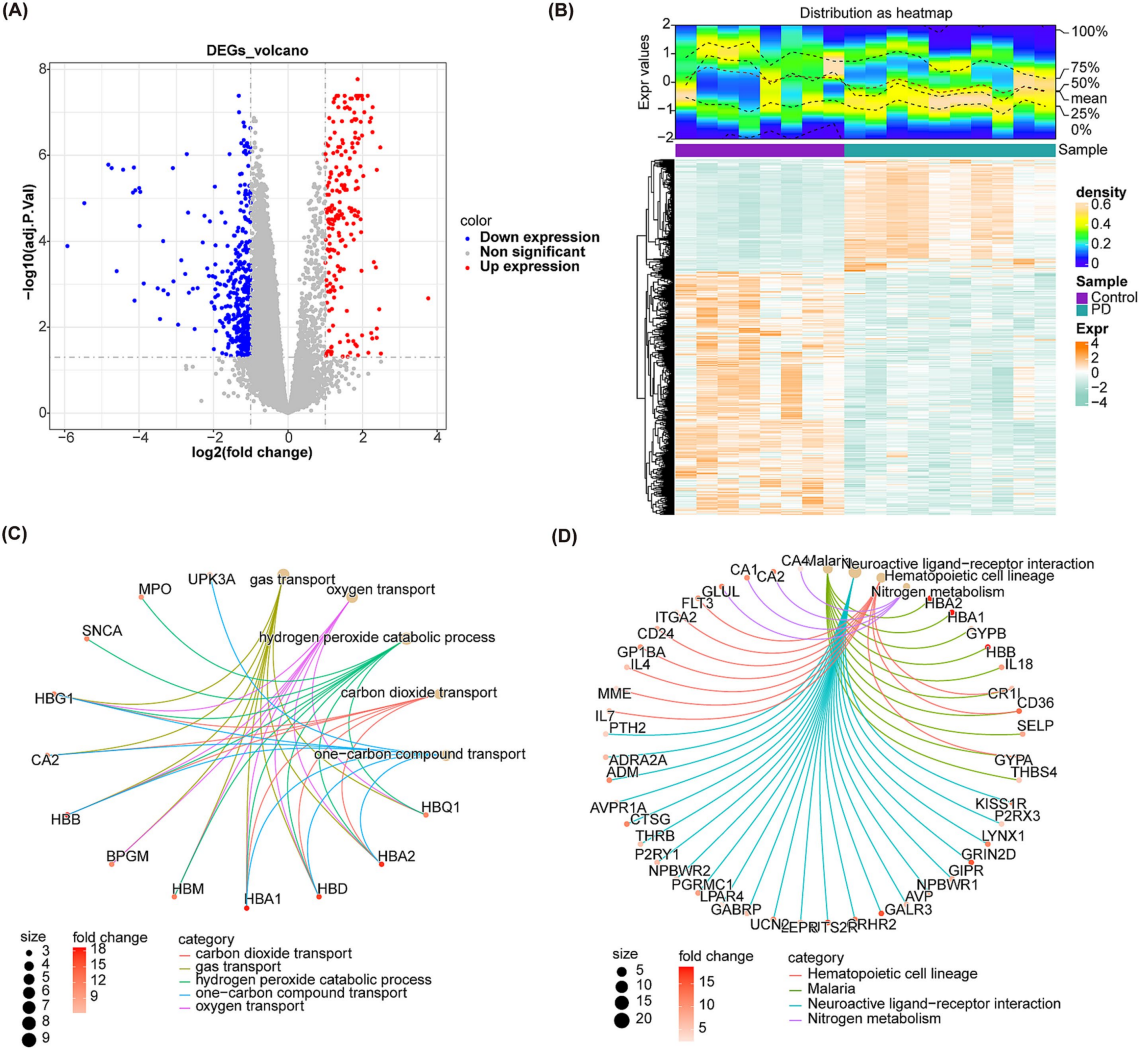
Identification of DEGs and enrichment of DEGs. **(A)** Volcano plot of differential expression analysis. The red dots represent up-regulated genes and the blue dots represent down-regulated genes. **(B)** Heatmaps of expression clusters for all genes in PD and control groups. Green represents down regulation, and orange represents up regulation. **(C)** The diagram shows GO enrichment of DEGs. **(D)** The diagram shows KEGG enrichment of DEGs.

**Figure 3:**
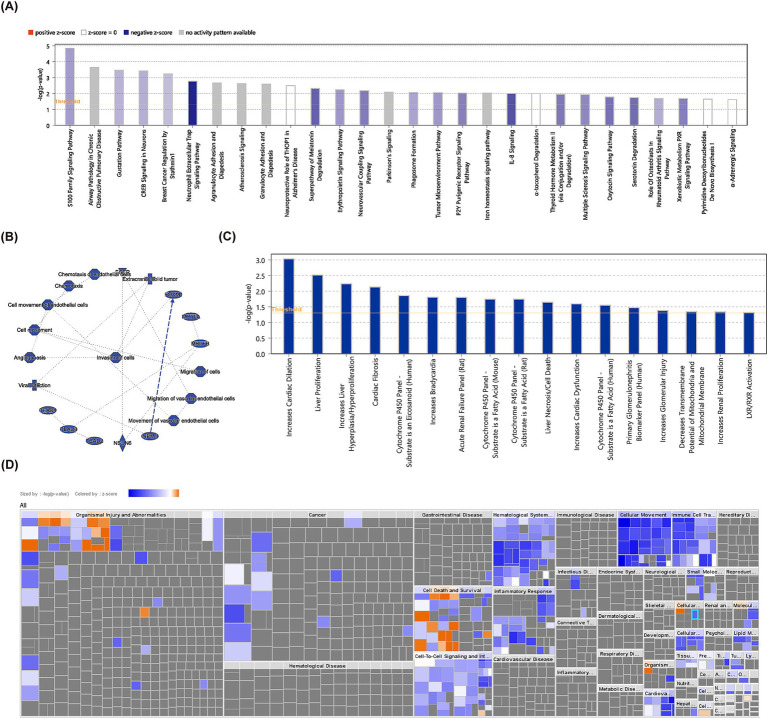
IPA analysis. **(A)** Classical pathway analysis using IPA in GSE22491 revealed enriched pathways for differentially expressed genes. **(B)** Biomarker regulatory network. **(C)** Disease function bar graph of differentially expressed genes in GSE22491. **(D)** Disease function items of differentially expressed genes.

A total of 634 DEGs were yielded between PD and control samples from GSE22491 (Supplementary material S1). Figure 2A shows the volcano map after the differential expression analysis of all genes. The heat map showed the expression and clustering of differentially expressed genes in PD group and control group (Figure 2B). Next, DEGs were analyzed for KEGG and GO enrichment, and the results showed that DEGs were involved in GO terms such as neutrophil-mediated killing of symbiont cell, regulation of vascular permeability, transmission of nerve impulse, regulation of transmission of nerve impulse (Figure 2C). Meanwhile, they were participated in four KEGG pathways containing hematopoietic cell lineage, nitrogen metabolism, neuroactive ligand-receptor interaction, Malaria (Figure 2D). Ultimately, IPA indicated that S100 family signaling pathway, gustation pathway, neutrophil extracellular trap signaling pathway, superpathway of melatonin degradation, erythropoietin signaling pathway and other classical pathways identified based on DEGs were found to be suppressed in PD (Figure 3A). Additionally, A Regulatory network of biomarkers was constructed, in which factors indirectly associated with biomarkers including Viral infection (disease-related), Cell movement (cell-related), angiogenesis (cell-related), NPM1 (TF), FOXM1 (TF), NSUN6 (gene), EGFR (chemical compound), etc., where NPM1 could indirectly inhibit FOXM1 (Figure 3B). Diseases and functional pathways identified based on DEGs including increases cardiac dilation, cardiac fibrosis, liver proliferation, increases liver hyperplasia/hyperproliferation, cytochrome p450 panel-substrate is an eicosanoid (human), etc., all of which were found to be suppressed in PD (Figures 3C,D).

### 3.2 Screening for key module

**Figure 4:**
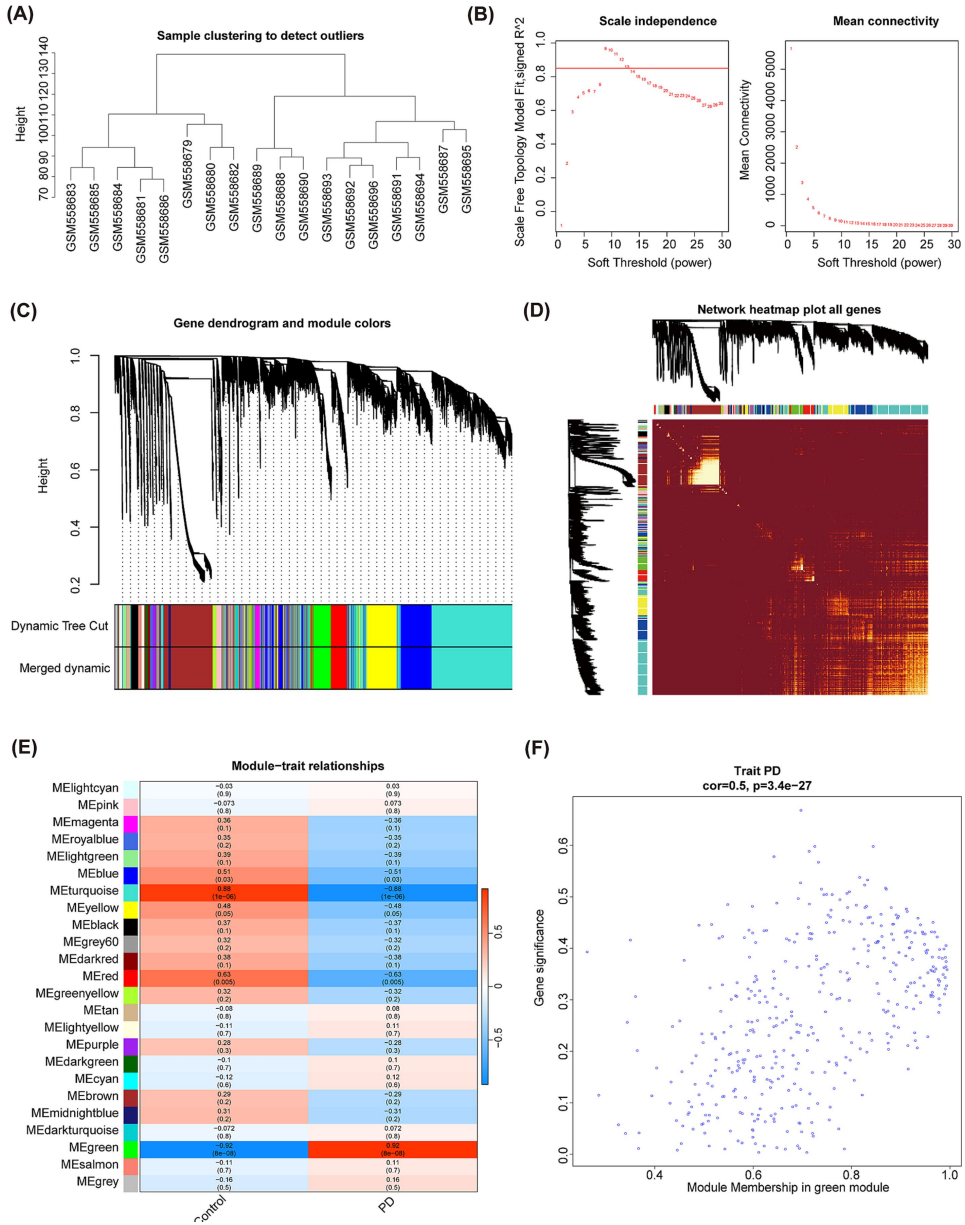
WGCNA analysis of GSE22491 and acquisition of key module. **(A)** Sample clustering tree diagram. **(B)** The selection of soft threshold power. **(C)** A tree diagram based on hierarchical clustering is generated. Different hues reflect different modules of co-expression. **(D)** Heatmap of correlation between different genes. **(E)** Correlation analysis between modules and PD clinical status. Blue represents negative correlation, and orange represents up positive correlation. A number inside a square shows the degree of statistical significance. **(F)** GS vs. MM correlation scatterplot of green module.

**Figure 5:**
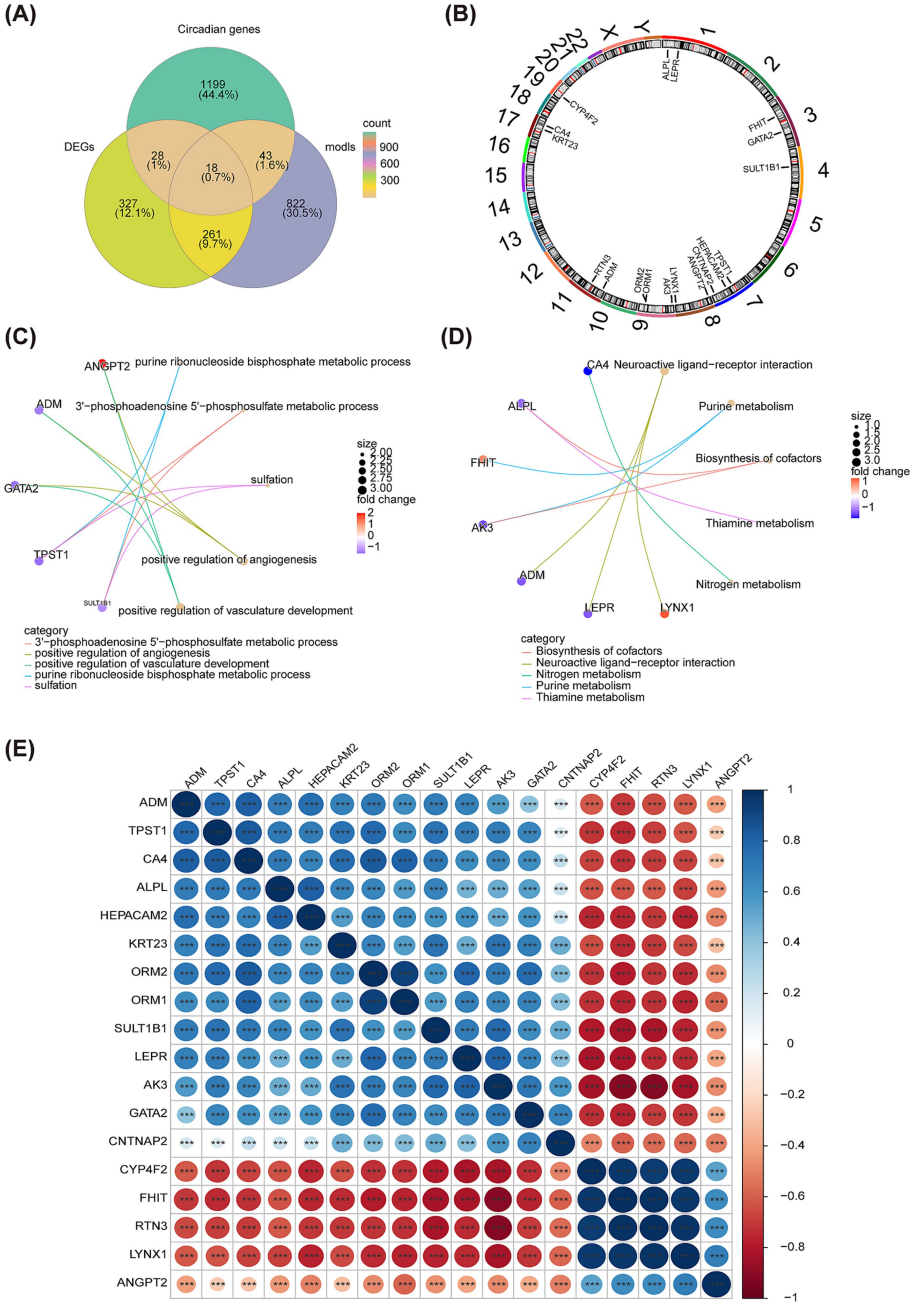
Identification of DECGs. **(A)** The Venn diagram shows the intersection of the green module genes of WGCNA and DEGs and C-genes. **(B)** The diagram shows the localization of DECGs on chromosomes. **(C)** The chordal graph shows GO enrichment of DECGs. **(D)** The chordal graph shows KEGG enrichment of DECGs. **(E)** Correlation heat map of DECGs. Red represents negative correlation, and blue represents positive correlation. *p < 0.05; **p < 0.01; ***p < 0.001; ****p < 0.0001.

Subsequently, the R package “WGCNA” was used to check the clustering outliers of the samples. The results show that general clustering of samples in GSE22491 was good, thus there was no need to exclude samples (Figure 4A). A soft threshold was chosen as 13 because vertical coordinate R^2 approached 0.85 at this time (Figure 4B). Next, totally 24 merged modules were finally sifted out (Figure 4C). Figure 4D shows the correlation heat maps of all the genes in these 24 modules. MEgreen module was highly correlated with PD (Cor = 0.92, and *p* < 0.05), therefore, it was defined as the key module containing 1,144 genes (Figure 4E). There is high correlation between Module Membership (MM) and Gene Significance (GS) (Cor = 0.5, and *p* < 0.05) (Figure 4F). Taking intersection of DEGs, C-genes and genes in the key module resulted in 18 DECGs (Figure 5A). Figure 5B illustrated localization of DECGs on chromosomes, where the most of genes were localized on chromosome 7, including TPST1, HEPACAM2 and CNTNAP2. Enrichment analyses showed that DECGs were enriched in GO entries, including positive regulation of angiogenesis, purine ribonucleoside bisphosphate metabolic process, and 3′ − phosphoadenosine 5′ − phosphosulfate metabolic process, etc. (Figure 5C; Supplementary material S2). They were also engaged in Neuroactive ligand−receptor interaction, Purine metabolism, Biosynthesis of cofactors and other KEGG pathways (Figure 5D; Supplementary material S3). Ultimately, there had notably negatively correlations among CYP4F2, FHIT, RTN3, LYNX1, and ANGPT2 (Figure 5E).

### 3.3 Biomarkers had terrific ability for distinguishing PD from control samples

**Figure 6:**
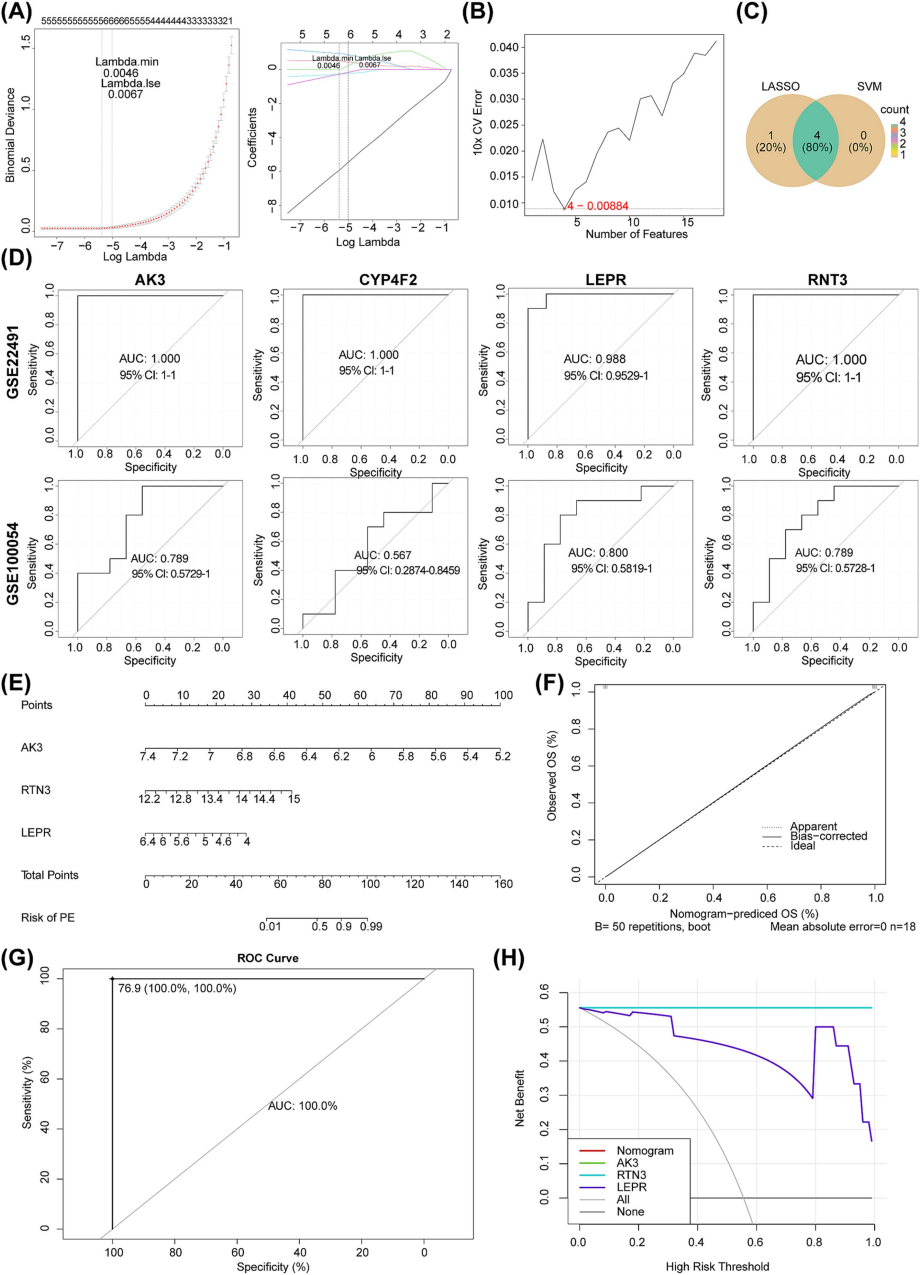
Identification of molecular biomarkers determined by machine learning and evaluation of their diagnostic value. **(A)** LASSO regression analysis. **(B)** SVM machine learning analysis. **(C)** The Venn diagram shows the LASSO regression analysis and SVM machine learning analysis results. **(D)** ROC curves of AK3, RTN3, CYP4F2 and LEPR in GSE22491 and GSE100054. **(E–H)** The nomogram, calibration curve, prediction model ROC curve and decision curve analysis of GSE22491 were performed.

To identify biomarkers, we screened 18 circadian rhythm-associated differential genes by the Lasso model, when lambda = 0.0046, error rate was the lowest, and five genes (AK3, RTN3, CYP4F2, LEPR, and HEPACAM2) were obtained at this point (Figure 6A). Four genes (AK3, RTN3, CYP4F2, and LEPR) were also yielded via SVM-RFE (Figure 6B). Four critical genes, namely AK3, RTN3, CYP4F2, and LEPR, were identified after taking intersection of genes obtained by LASSO and SVM-RFE (Figure 6C). In GSE22491 and GSE100054, AUC values of AK3, RTN3 and LEPR all exceeded 0.7, indicating that they had terrific ability for distinguishing PD from control samples, thus they were defined as biomarkers (Figure 6D). Corners of ROC curve indicated optimal critical value point, with the values in parentheses Specificity and Sensitivity, respectively. It could be seen that sensitivity and specificity of optimal critical value points in all ROC curves were high [AK3 (1,1), RTN3 (1,1), CYP4F2 (1,1), and LEPR (1,0.9)], and false positives and false negatives were also the least, which indicated that AUC of ROC curve was the most desirable examination index at this time. Additionally, C-index of nomogram was 1, suggesting that diagnostic model had a nice ability for predicting PD patients. In calibration curve, “Apparent,” “Bias−corrected,” and “Ideal” curves were almost coincident, and their slopes were closed to 1, suggesting that diagnostic model could prediced PD patients accurately, AUC of diagnostic model was 1 further validated that it could prediced PD patients well, DCA curve showed that nomogram curve was higher than ALL and None curves, indicating that the diagnostic model could be applied to PD patients (Figures 6E–H).

### 3.4 Biomarkers were associated with PD related pathways

**Figure 7:**
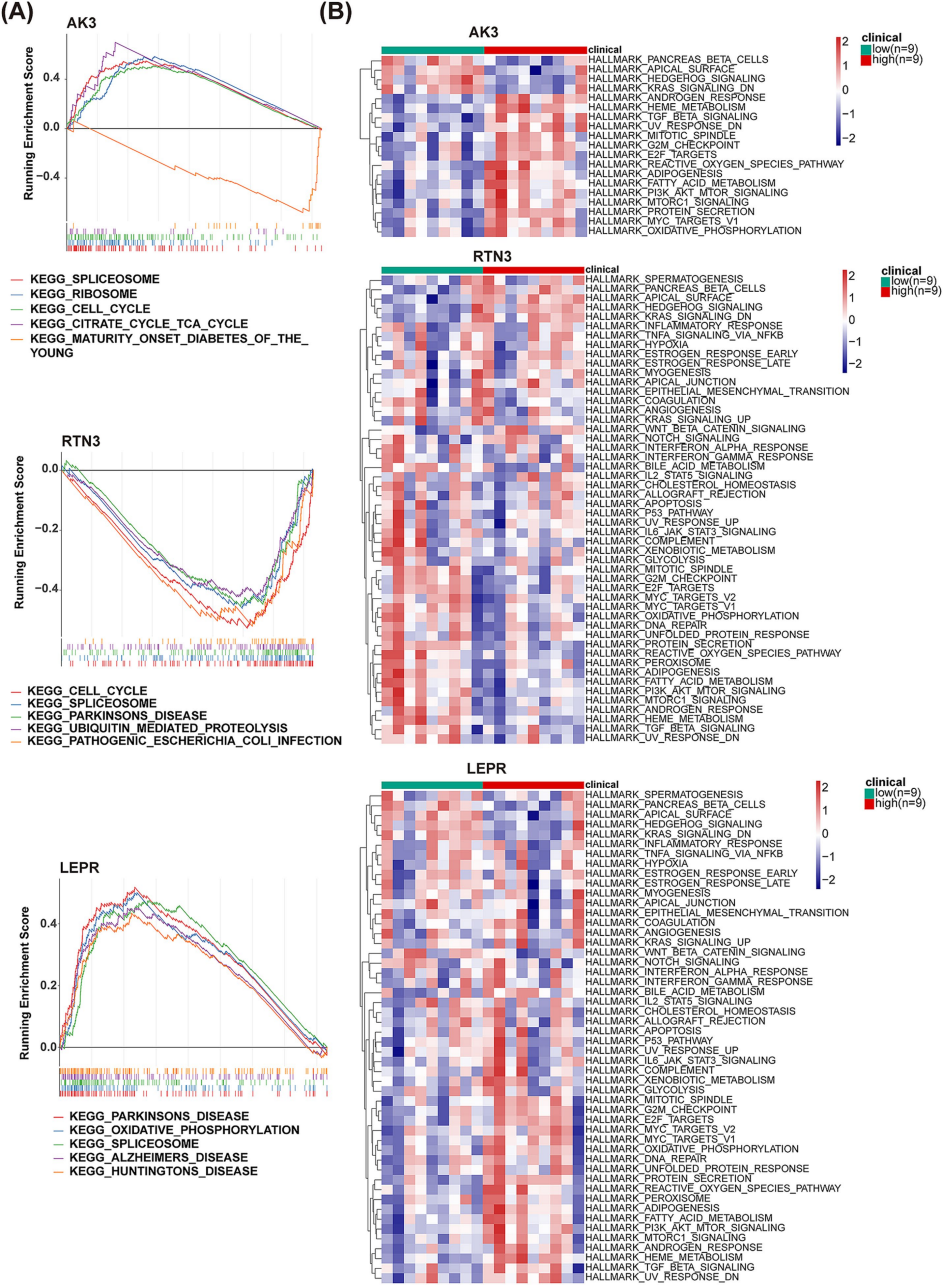
GSEA and GSVA analysis of AK3, RTN3 and LEPR. **(A)** GSEA analysis results of AK3, RTN3 and LEPR. **(B)** GSVA analysis heatmap of AK3, RTN3 and LEPR.

For exploring functional pathways involved in biomarkers, GSEA was carried out. KEGG enrichment results showed that AK3 positively regulated SPLICEOSOME, RIBOSOME, CELL_CYCLE and CITRATE CYCLE TCA_CYCLE pathways and negatively regulated MATURITY ONSET DIABETES OF THE_YOUNG pathway. The RTN3 negatively regulated the CELL_CYCLE, SPLICEOSOME, PARKINSONS_DISEAS, UBIQUITIN_MEDIATED PROTEOLYSIS and PATHOGENIC ESCHERICHIA_COLLINFECTION pathways. The LEPR positively regulated the PARKINSONS DISEASE, OXIDATIVE_PHOSPHORYLATION, SPLICEOSOME, ALZHEIMERS DISEASE and HUNTINGTONS DISEASE pathways (Figure 7A). Meanwhile, pancreas beta cells, apical surface, hedgehog signaling, kras signaling, heme metabolism, oxidative phosphorylation and other hallmark pathways were simultaneously and differentially enriched in high/low expression groups for each biomarker (Figure 7B).

### 3.5 Biomarkers was important for PD’s immune microenvironment

**Figure 8:**
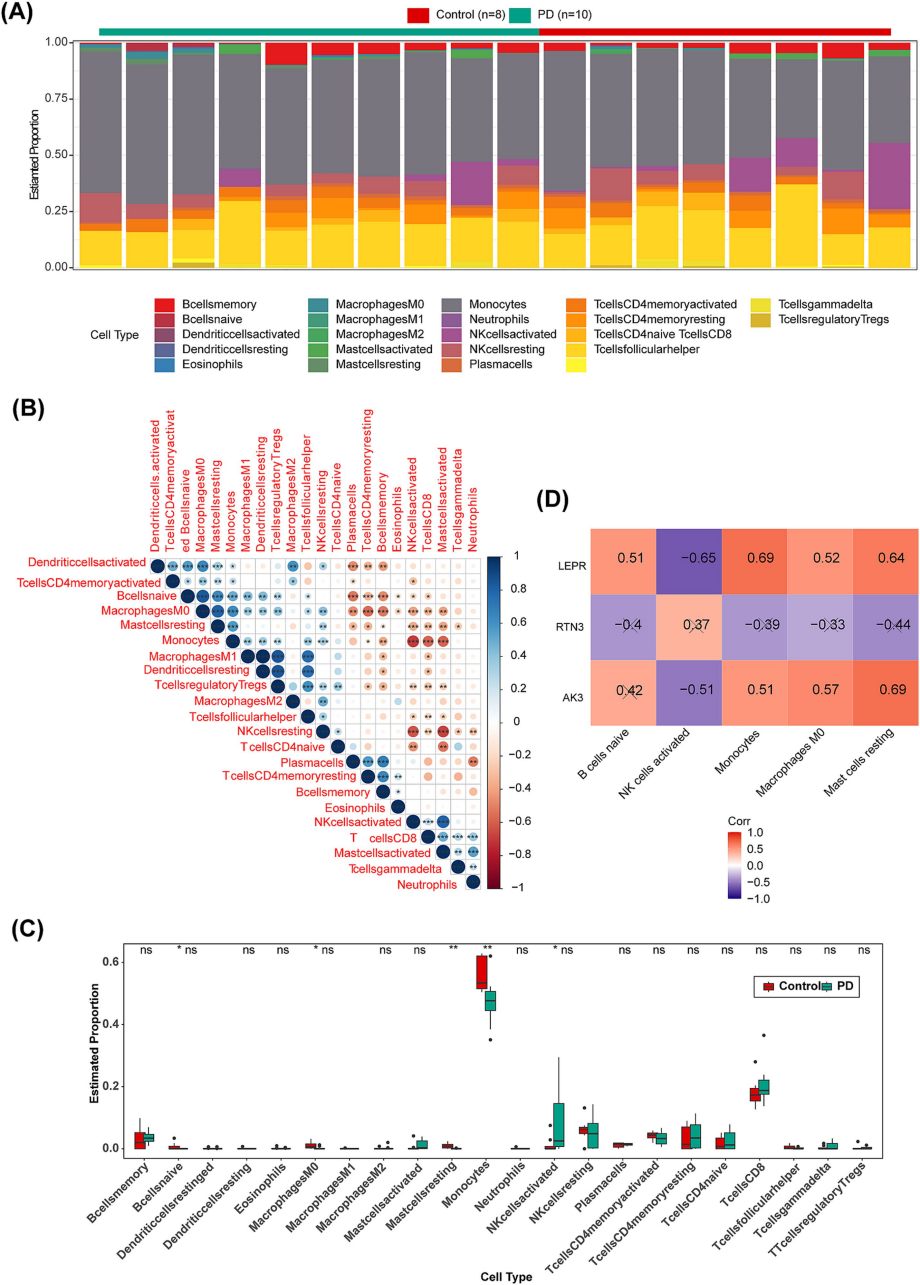
Immune infiltration analysis of GSE22491. **(A)** Distribution of 22 kinds of immune cells in PD and control groups. **(B)** Correlation analysis and heat map of 22 immune cells. Red represents negative correlation, and blue represents positive correlation. **(C)** Boxplot of infiltration of 22 immune cells in PD and control groups. **(D)** Heat map for correlation analysis of biomarker (AK3, RTN3 and LEPR) and five immune cells (B cells naïve, NK cells activated, Monocytes, Macrophages M0 and Mast cells resting). “×“means statistically significant. *p < 0.05; **p < 0.01; ***p < 0.001; ****p < 0.0001; ns = no significance.

Next, we used CIBERSORT to assess the relative abundance of 22 immune cells per sample in the training set GSE22491. It could be seen from the stacked plot that the monocytes occupied a relatively high proportion in GSE22491 samples (Figure 8A). Besides, the highest positive correlation was found between M1 macrophages and resting dendritic cells (Cor = 1, *p* < 0.05) (Figure 8B). The box plot revealed notable differences in the proportion of M0 macrophages, naive B cells, activated NK cells, resting mast cells, monocytes, between PD and control samples, except the activated NK cells, the other four immune cell types were highly expressed in control samples (Figure 8C). Apparently, LEPR was strongly negatively correlated with activated NK cells (Cor = −0.65, *p* < 0.05) (Figure 8D).

### 3.6 The GeneMANIA, ceRNA, and mRNA-miRNA-TF networks

**Figure 9:**
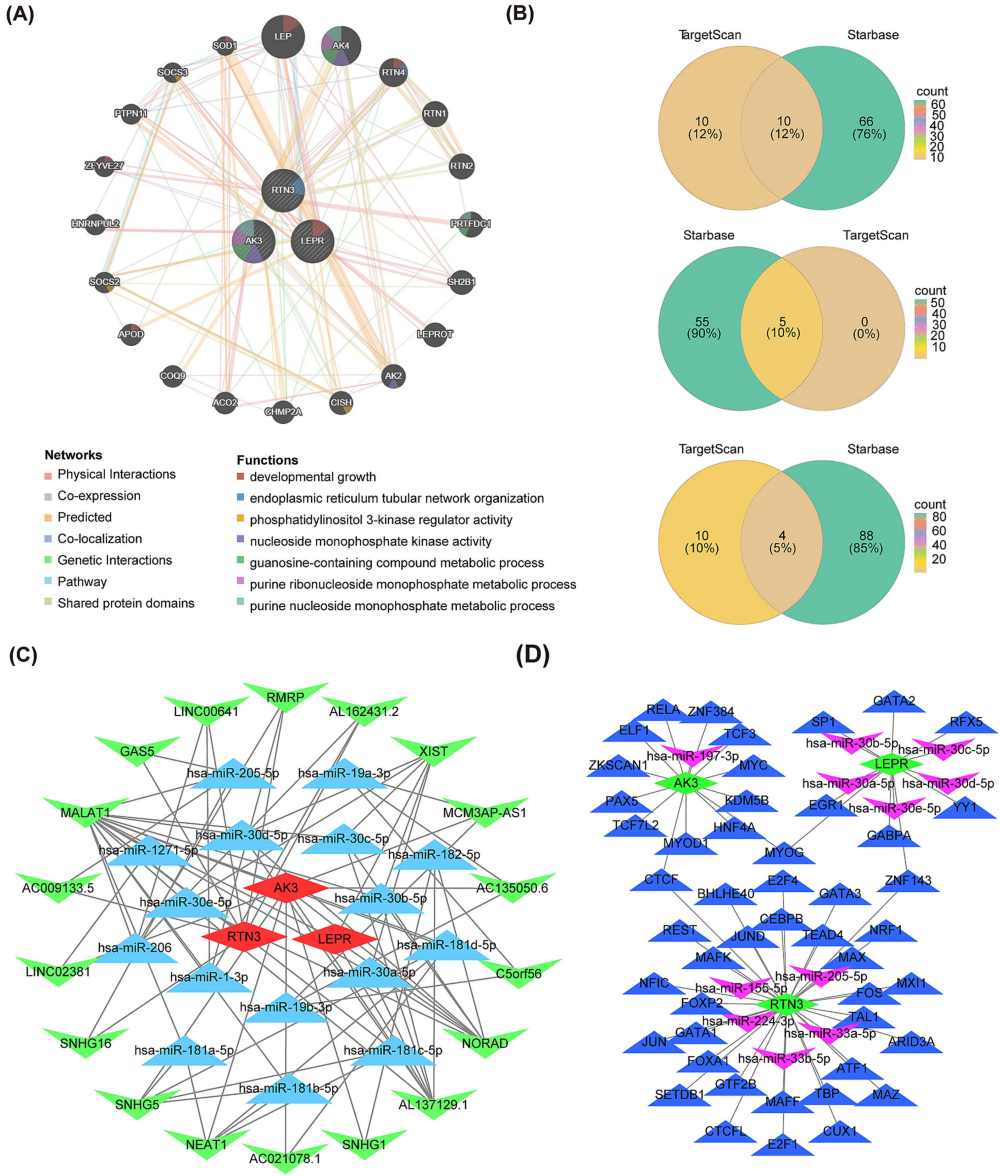
Interaction network of AK3, RTN3 and LEPR. **(A)** Gene–gene interaction network of AK3, RTN3 and LEPR. **(B)** Venn diagram shows the common miRNA of AK3, RTN3 and LEPR in the TargetScan and Starbase databases. **(C)** The ceRNA regulatory network of AK3, RTN3 and LEPR. Red is the biomarkers; blue is the miRNAs and green is the lncRNAs. **(D)** mRNA-miRNA-TF network of AK3, RTN3 and LEPR. Biomarkers are shown in green, miRNA in purple, and TF in blue.

A total of 20 genes associated with biomarkers were predicted through GeneMANIA database, and they were involved in 7 pathways. Among these pathways, LEP, RTN4, APOD, ZFYVE27, SOD1, and LEPR collectively played a role in developmental growth (Figure 9A). AK3, LEPR, and RNT3, respectively, correspond to 10, 5, and 4 intersected miRNAs targeting them (Figure 9B). In the ceRNA network, there was a phenomenon where one lncRNA simultaneously regulated multiple miRNAs at the same time was existed. For example, MALAT1 could simultaneously target hsa-miR-1-3p, hsa-miR-205-5p, etc., while NEAT1 could simultaneously target hsa-miR-181a-5p, hsa-miR-181b-5p, etc. (Figure 9C). The ceRNA network related information of the three molecular markers is shown in Table 2. Besides, AK3, LEPR, and RNT3 were, respectively, corresponded to 13 TFs, 8 TFs, and 30 TFs (Table 3). Among these TFs, CTCF could regulate both AK3 and RNT3, MYOG could simultaneously regulate AK3 and LEPR, and ZNF143 could regulate LEPR and RNT3 at the same time. Furthermore, an mRNA-miRNA-TF network containing relationship pairs such as PAX5-AK3, GATA2-LEPR, FOXP2-RTN3 was constructed (Figure 9D).

**Table 2:** The ceRNA network information of RTN3, LEPR, and AK3.

mRNA	miRNA	lncRNA			
RTN3	hsa-miR-205-5p	GAS5	MALAT1	AC009133.5	SNHG16
	hsa-miR-206	AL162431.2	RMRP	MALAT1	LINC00641
	hsa-miR-1-3p	AL162431.2	RMRP	MALAT1	LINC00641
	hsa-miR-218-5p	No lncRNA target			
LEPR	hsa-miR-30e-5p	MALAT1	AL137129.1	NORAD	XIST
	hsa-miR-30d-5p	MALAT1	AL137129.1	NORAD	XIST
	hsa-miR-30a-5p	MALAT1	AL137129.1	NORAD	XIST
	hsa-miR-30b-5p	MALAT1	AL137129.1	NORAD	XIST
	hsa-miR-30c-5p	MALAT1	AL137129.1	NORAD	XIST
AK3	hsa-miR-1271-5p	MALAT1	LINC02381		
	hsa-miR-182-5p	SNHG1	AL137129.1	NORAD	
	hsa-miR-186-5p	No lncRNA target			
	hsa-miR-340-5p	No lncRNA target			
	hsa-miR-181c-5p	SNHG5	NEAT1	MALAT1	
	hsa-miR-181a-5p	SNHG5	NEAT1	MALAT1	
	hsa-miR-181b-5p	SNHG5	NEAT1	MALAT1	
	hsa-miR-181d-5p	AC021078.1	SNHG5	NEAT1	MALAT1
	hsa-miR-19a-3p	C5orf56	AC135050.6	MCM3AP-AS1	
	hsa-miR-19b-3p	C5orf56	AC135050.6	MCM3AP-AS1	

**Table 3:** Gene and transcription factors (TFs).

Gene	TFs
AK3	TCF7L2, PAX5, ZKSCAN1, ELF1, RELA, ZNF384, TCF3, MYC, KDM5B, HNF4A, MYOD1, MYOG, CTCF
LEPR	EGR1, SP1, GATA2, RFX5, YY1, GABPA, MYOG, ZNF143
RTN3	FOXP2, MAFK, JUND, CEBPB, NFIC, REST, TEAD4, MAX, BHLHE40, E2F4, GATA3, NRF1, FOS, MXI1, TAL1, ARID3A, ATF1, TBP, MAFF, MAZ, CUX1, E2F1, GTF2B, FOXA1, CTCFL, SETDB1, GATA1, JUN, CTCF, ZNF143

### 3.7 Prediction of potential therapeutic agents

**Figure 10:**
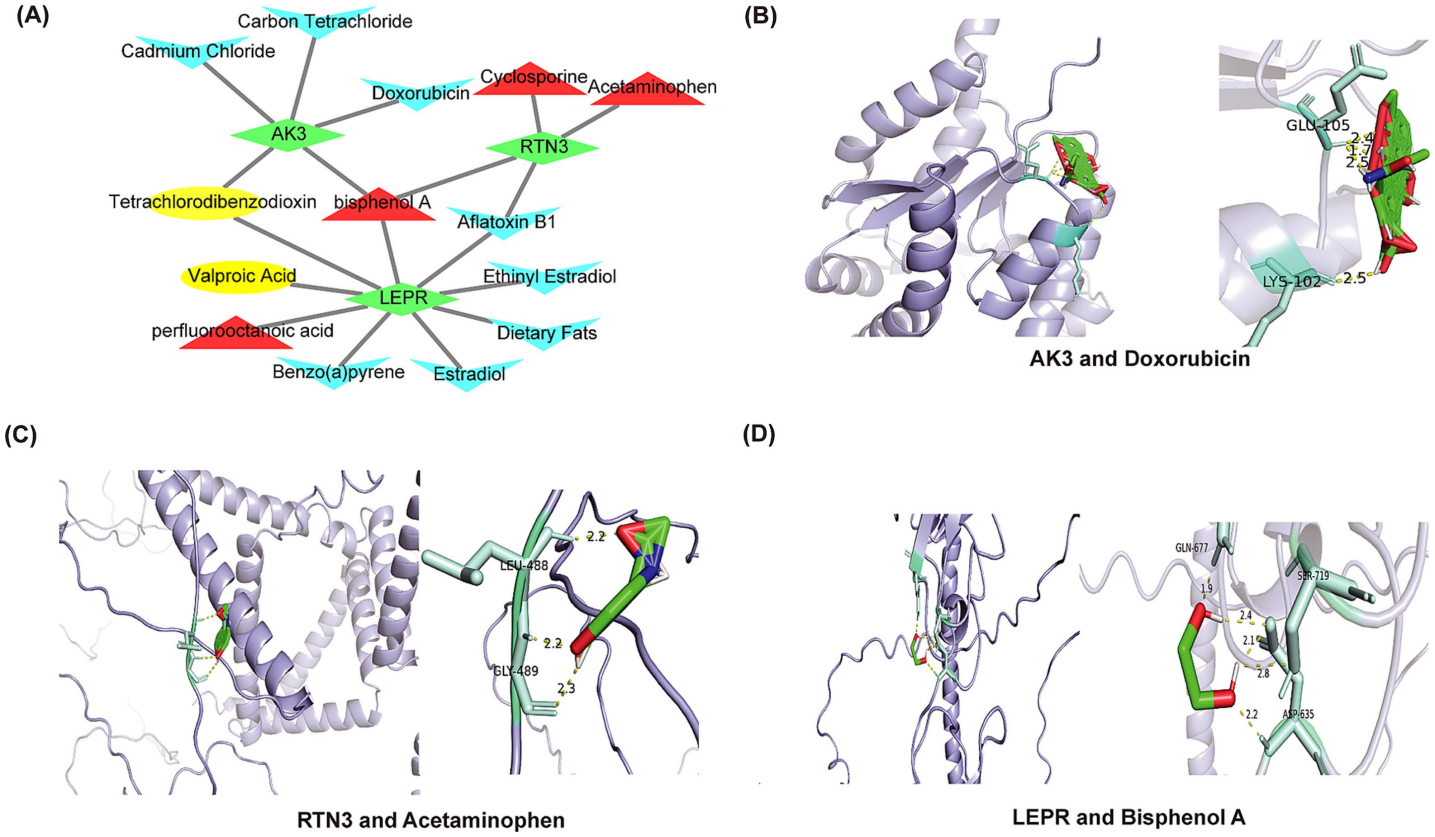
Prediction of drugs that affect biomarkers expression and molecular docking. **(A)** The network interaction map showed the drug target prediction. Biomarkers are shown in green. Drugs that upregulate target genes are represented in red; drugs that downregulate target genes are represented in blue; and drugs that affect target genes are represented in yellow. **(B)** Molecular docking of AK3 and doxorubicin. **(C)** Molecular docking of RTN3 and acetaminophen. **(D)** Molecular docking of LEPR and bisphenol A.

The 14 potential therapeutic agents were identified based on biomarkers, each of which had different effects on biomarkers expression. For instance, bisphenol A increased expression of AK3, RTN3 and LEPR, while Aflatoxin B1 inhibited expression of both RTN3 and LEPR. A drug-gene network was constructed, including relationship pairs such as cadmium Chloride-AK3, Estradiol-LEPR, Cyclosporine-RTN3 and other relationship pairs was constructed (Figure 10A). Eventually, AK3 was molecularly docked with doxorubicin. Forming two hydrogen bonds including GLU-105 and LYS-102 (Figure 10B). RNT3 was docked to acetaminophen, forming two hydrogen bonds (LEU-488 and GLY-489) (Figure 10C). LEPR was molecularly docked to bisphenol A by three hydrogen bonds including GLN-677, SER-719 and ASP-635 (Figure 10D). The molecular docking energies of AK3 and doxorubicin, RTN3 and acetaminophen, and LEPR and bisphenol A were − 4.04 kcal/mol, −3.14 kcal/mol, and − 3.735 kcal/mol, respectively.

### 3.8 Gene expression levels in GEO data sets and qRT-PCR experiments

**Figure 11:**
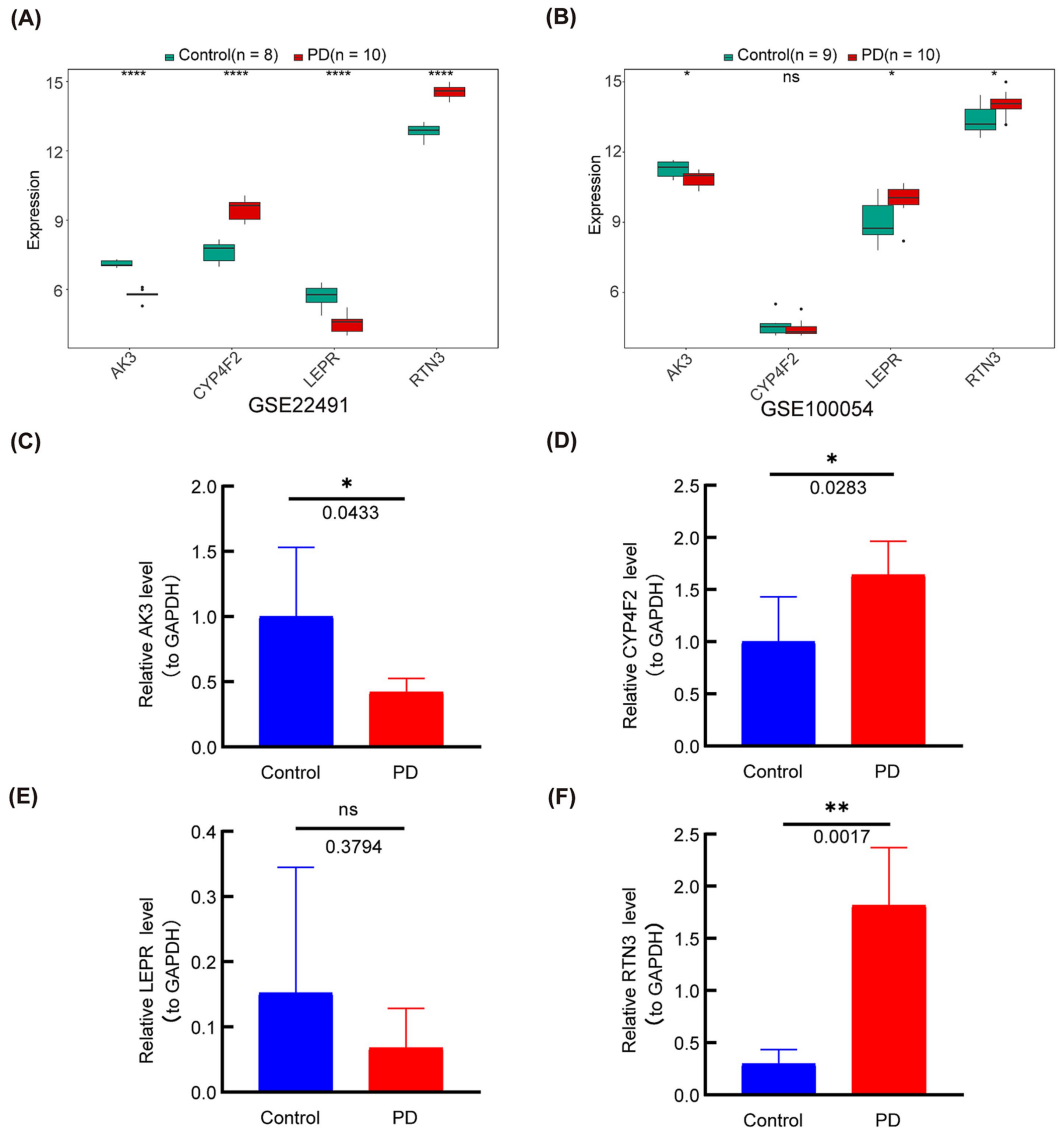
The expression levels of the four genes (AK3, CYP4F2, LEPR and RTN3) were analyzed in the GEO datasets and verified by q-PCR. (A) The expression level of genes in GSE22491. **(B)** The expression level of genes in GSE100054. **(C)** Gene expression level of AK3 was determined by q-PCR. **(D)** Gene expression level of CYP4F2 was determined by q-PCR. **(E)** Gene expression level of LEPR was determined by q-PCR. **(F)** Gene expression level of RTN3 was determined by q-PCR. *p < 0.05; **p < 0.01; ***p < 0.001; ****p < 0.0001; ns = no significance.

To demonstrate the expression levels of the biomarkers in GSE22491 and GSE100054, we performed qRT-PCR experiments. In GSE22491, AK3 and LEPR in PD patients were significantly down-regulated, while CYP4F2 and RTN3 were significantly up-regulated (Figure 11A). In GSE100054, AK3 was down-regulated in PD patients, while LEPR and RTN3 were significantly up-regulated (Figure 11B). In the qRT-PCR test, AK3 was down-regulated (*p* = 0.0433) and RTN3 up-regulated (*p* = 0.0283) in PD, and the expression was consistent with the results in GEO datasets. CYP4F2 was up-regulated in PD, and LEPR was not differentially expressed. The qRT-PCR results are shown in Figures 11C–F.

## 4 Discussion

Parkinson’s disease is the second most common neurodegenerative disease, and its incidence is increasing year by year (Emamzadeh and Surguchov, 2018). PD-related symptoms have a serious impact on the normal life of patients and their families. The current diagnosis of PD is based on clinical criteria that require the presence of retardation along with at least one motor symptom, such as tremor, stiffness, or postural instability (Tolosa et al., 2021). However, the latency period from the first non-motor symptoms of Parkinson’s disease to meeting the current diagnostic criteria for PD can range from 5 to 20 years, and we also call this period the prodromal phase of PD. Through the study of body fluid and tissue biomarkers, clinical non-motor symptoms and neuroimaging related to PD prodromal stage, it can provide new ideas for relieving and preventing PD motor symptoms. Many processes in the human body, including brain function, are regulated over a 24-h cycle, and there is a strong association between circadian rhythm disturbances, such as sleep–wake cycles, and central nervous system disorders. Studies have shown that 80% of PD patients suffer from sleep–wake disorders, and nighttime hypertension is almost universal in PD patients, and patients’ blood pressure often reverses, with nighttime blood pressure higher than daytime levels (Hunt et al., 2022). Although various circadian rhythm real-time detection instruments have been used to observe the rhythm disturbance in PD patients, the mechanism of circadian rhythm disturbance in PD patients and the impact of circadian rhythm disturbance on the occurrence and development of PD are not clear.

**Figure 12:**
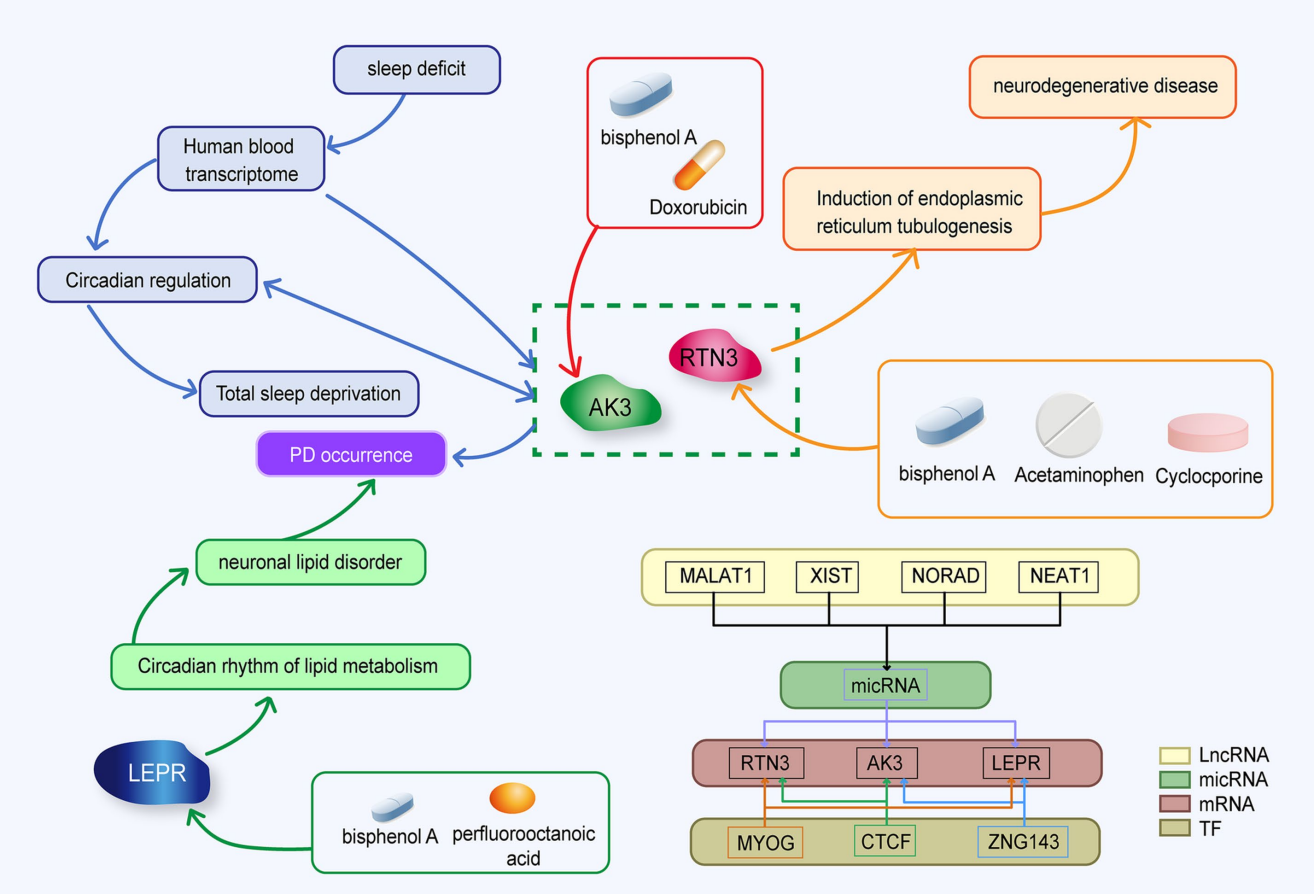
Mechanistic diagram of circadian rhythm-related molecular markers.

The purpose of this study was to screen out blood molecular markers related to prodromal Parkinson’s disease on the one hand, and to explore the relationship and mechanism between circadian rhythm disturbance and PD development on the other hand. We analyzed the expression of circadian rhythm-related genes and the enrichment of functional pathways in PD patients by bioinformatics. In addition, we used machine learning methods to screen out candidate markers with diagnostic value, and used other data sets to conduct external validation with clinical patient blood samples. Elize Aparecida Santos Musachio et al. found that bisphenol A can cause changes in drosophila Parkinson’s disease through oxidative stress (Musachio et al., 2020). Drug target prediction indicated that bisphenol A could up-regulate the expression of LEPR, AK3 and RTN3 (Ptak and Gregoraszczuk, 2012; Thongkorn et al., 2019; Ooi et al., 2021). The interaction of the three molecular markers is shown in Figure 12.

The leptin protein receptor, encoded by the LEPR gene, act as a regulator of energy homeostasis and feeding behavior (Yin X. et al., 2022; Yin Z. et al., 2022). Adipose tissue, a metabolically active organ, showing highly rhythmic behavior. MADRID et al. found that the expression of LEPR, which is related to fat metabolism, showed obvious rhythmic behavior (Garaulet et al., 2011). In contrast, Wang et al. found that the expression of the LEPR gene was significantly elevated in PD patients compared to healthy controls (Yin X. et al., 2022; Yin Z. et al., 2022). This contradicts our results. Leptin maintains cell survival in neuronal SH-SY5Y cells by maintaining ATP levels and mitochondrial membrane potential to resist MPP^+^ toxicity (Parkinson’s disease model) (Shin et al., 2011). The up-regulation of the LEPR gene may indicate that neurons activate feedback protection or that early downregulation of LEPR activates a cascade that promotes upregulation. Analyzing the expression of LEPR in different stages of PD development is of great significance for exploring the occurrence, development, and etiology of PD.

Reticulon 3 (RTN3), which is a member of the reticulon family of proteins, that is preferentially expressed in neuroendocrine tissues. Additionally, it has been discovered that RTN3 interacts with beta-amyloid converting enzyme 1 (BACE1), an enzyme that starts the production of amyloid peptides from amyloid precursor protein and reticulon proteins are negative modulators of BACE1 in cells (Sharoar and Yan, 2017). Sleep deprivation affects transcriptome levels in the blood, disrupting their circadian regulation and exacerbating the effects of acute total sleep deprivation. RTN3 is one of the genes affected by circadian rhythms (Möller-Levet et al., 2013). Studies have revealed that RTN3 is a particular receptor for the breakdown of endoplasmic reticulum tubules (Grumati et al., 2017). RTN3 belongs to the reticulin family, which is most abundant in the brain, and it is frequently activated in response to endoplasmic reticulum stress. Down-regulation of RTN3 has been demonstrated to enhance the clearance of cytoplasmic PrP aggregates, restore the activity of the ubiquitin proteasome system, and reduce endoplasmic reticulum stress, and the clearance of cytoplasmic PrP aggregates has been shown to suppress apoptosis. Therefore, the targeted suppression of RTN3 expression helps to lower the stress level of neuronal cells and stop the progression to apoptosis (Chen et al., 2011). In this study, the expression of RTN3 was up-regulated in PD patients, which may serve as a potential target for future treatment of organelle oxidative stress and inhibition of neuronal apoptosis in PD patients. Some studies have found that PD patients have more peripheral natural killer (NK) cells than non-PD controls, and Rachael H Earls et al. demonstrated that NK cells successfully internalize and destroy *α*-syn aggregates via the endosome/lysosome route (Earls and Lee, 2020; Earls et al., 2020). In this study, immunoinfiltration analysis revealed that the number of activated NK cells in the peripheral blood of Parkinson’s disease patients was greater than in the control group. This finding proves and supplements the results of prior investigations. In the correlation analysis between biomarkers and immune cells, RTN3 showed a high positive correlation with activated NK cells. The increased expression of RTN3 in PD patients may be a significant target in the process of encouraging the aggregation of NK cells, offering a fresh perspective on how α-syn is degraded. Sacha Bohler et al. have suggested that acetaminophen overdose is a potential risk factor for Parkinson’s disease (Bohler et al., 2019). Acetaminophen can up-regulate the expression of RTN3 (Yu et al., 2018). Aya Yassin Labib et al. found that acetaminophen had a neuroprotective effect on rotenone-induced Parkinson’s disease in rat models (Labib et al., 2021). Different doses of acetaminophen may have different effects on PD, which requires us to find out the answer in further large-scale clinical studies and related experiments.

Adenylate Kinase 3 (AK3) is a Protein Coding gene. Diseases associated with AK3 include Reticular Dysgenesis and Orofacial Cleft 8. AK3 functions to participate in the maintenance of cellular nucleotide homeostasis by catalyzing interconversion between nucleotides. Down-regulation of genes in the substantia nigra may be regarded as a result of dopaminergic cell death. Paula Garcia-Esparcia et al. discovered that the expression of AK3 was down-regulated in the substantia nigra at stages 3–6 (Garcia-Esparcia et al., 2015). In GEO database and q-PCR verification, AK3 was down-regulated in PD groups. Doxorubicin results in decreased expression of AK3 mRNA (Vijay et al., 2016; Verheijen et al., 2018). A prominent feature of Parkinson’s disease is α-syn protein aggregation, and there have been cases where patients undergoing Doxorubicin are prone to Parkinson-like symptoms. It may be due to protein aggregation caused by Doxorubicin (Garg and Sinha, 2022).

In this study, by means of GSVA and GSEA enrichment analysis, three core biomarkers were significantly enriched in key biological processes such as OXIDATIVE_PHOSPHORYLATION, DNA_REPLICATION and DNA_REPAIR. Some studies have confirmed that the oxidative phosphorylation activity of rat brain mitochondria is regulated by rotenone and melatonin, showing a significant circadian rhythm (Spira and Spiegel, 1992). This finding not only highlights the central role of mitochondrial function in the pathological mechanism of PD, but also reveals the underlying circadian regulation mechanism. At the same time, it is particularly worth mentioning that the regulatory effect of melatonin on health and disease states has also been fully confirmed (Reiter et al., 2021). Studies have shown that melatonin can promote the transition from aerobic glycolysis to mitochondrial oxidative phosphorylation in solid tumor cells and other pathological cells (including Alzheimer’s disease, fibrosis lesions, macrophage hyperactivation, etc.). This mechanism may be the cornerstone of its inhibition of disease development and maintenance of health. In addition, the roles of key enzymes in DNA repair and replication, as well as their important roles in the pathogenesis of neurodegenerative diseases, have also been investigated (Catarzi et al., 2022). The changes in the activities of these enzymes not only reflect the stability of the DNA repair system, but also may be used as biomarkers for the early diagnosis of neurodegenerative diseases such as PD, providing a new perspective and direction for the early intervention and treatment strategies of the disease. Taken together, these findings not only enrich our understanding of the pathogenesis of PD and other neurodegenerative diseases, but also provide valuable scientific evidence for the development of future disease diagnosis and treatment strategies, especially for the exploration of new therapeutic targets based on mitochondrial function and DNA repair systems.

In our study, three key genes related to circadian rhythm were identified by bioinformatics analysis, which were AK3, RTN3 and LEPR. In addition, we constructed a diagnostic prediction model and proved that the model had good diagnostic prediction ability. Functional enrichment showed that these three biomolecular markers played an important role in the pathogenesis and development of PD. Analysis of immune infiltration AK3, RTN3 and LEPR may be involved in the progression of PD by regulating NK cells. However, how these genes contribute to PD progression by influencing related genes still needs to be further investigated in clinical trials. The mechanism of the influence of the three genes on circadian rhythm needs to be further studied in the experiment. These key genes may serve as new biomarkers of PD in the future and provide more references for the exploration of the pathogenesis of PD in the future. And we will continue to pay attention to the research progress of circadian rhythm and Parkinson’s disease.

There are some limitations to this study. First, this study mainly uses bioinformatics analysis to process transcriptome data in public databases, and although the gene-chip results may be flawed, the data used is rigorously processed and screened. Since this study is a preliminary exploration, a small sample size is used for rapid verification, and the results will provide a basis for subsequent large-scale studies. Although this study had a small sample size and was not disaggregated by sex, we have taken various measures to ensure the accuracy and reliability of the data. Secondly, we realized that the sample size of the current study was limited, so we tried different data sets for verification, and verified by experimental means. The combination of the three obtained a credible result to further ensure its potential application value. Finally, since the current AUC value of the model is 0.7, although it shows certain predictive ability, in order to ensure the applicability of the model in clinical practice, we recognize that a larger number of samples are needed for further experiments and validation. In the future, we will increase the study sample size, classify the sexes, and add more experimental validation, such as protein level verification, gene editing experiments, etc., to ensure the robustness and generalization ability of the model.

## Data Availability

The raw data supporting the conclusions of this article will be made available by the authors, without undue reservation.

## Footnotes

^1^https://www.ncbi.nlm.nih.gov/gds

^2^http://cgdb.biocuckoo.org

^3^http://starbase.sysu.edu.cn/

^4^https://www.targetscan.org/vert_80/

^5^https://maayanlab.cloud/chea3/

^6^http://ctdbase.org/

^7^https://www.rcsb.org/

^8^https://pubchem.ncbi.nlm.nih.gov/

^9^https://www.r-project.org/

## References

[ref1] Aarsland D. Batzu L. Halliday G. M. Geurtsen G. J. Ballard C. Ray Chaudhuri K.. (2021). Parkinson disease-associated cognitive impairment. Nat. Rev. Dis. Primers 7:47. doi: 10.1038/s41572-021-00280-3 34210995

[ref2] Bérard M. Sheta R. Malvaut S. Rodriguez-Aller R. Teixeira M. Idi W.. (2022). A light-inducible protein clustering system for in vivo analysis of α-synuclein aggregation in Parkinson disease. PLoS Biol. 20:e3001578. doi: 10.1371/journal.pbio.3001578, PMID: 35263320 PMC8936469

[ref3] Berman H. M. Westbrook J. Feng Z. Gilliland G. Bhat T. N. Weissig H.. (2000). The Protein Data Bank. Nucleic Acids Res. 28, 235–242. doi: 10.1093/nar/28.1.235, PMID: 10592235 PMC102472

[ref4] Bohler S. Liu X. Krauskopf J. Caiment F. Aubrecht J. Nicolaes G.. (2019). Acetaminophen overdose as a potential risk factor for Parkinson's Disease. Clin. Transl. Sci. 12, 609–616. doi: 10.1111/cts.12663, PMID: 31305025 PMC6853143

[ref5] Catarzi D. Varano F. Vigiani E. Lambertucci C. Spinaci A. Volpini R.. (2022). Casein kinase 1δ inhibitors as promising therapeutic agents for neurodegenerative disorders. Curr. Med. Chem. 29, 4698–4737. doi: 10.2174/0929867329666220301115124, PMID: 35232339

[ref6] Chang K. H. Chen C. M. (2020). The role of oxidative stress in Parkinson's Disease. Antioxidants (Basel) 9:597. doi: 10.3390/antiox9070597, PMID: 32650609 PMC7402083

[ref7] Chen R. Jin R. Wu L. Ye X. Yang Y. Luo K.. (2011). Reticulon 3 attenuates the clearance of cytosolic prion aggregates via inhibiting autophagy. Autophagy 7, 205–216. doi: 10.4161/auto.7.2.14197, PMID: 21119307

[ref8] Davis A. P. Grondin C. J. Johnson R. J. Sciaky D. Wiegers J. Wiegers T. C.. (2021). Comparative Toxicogenomics database (CTD): update 2021. Nucleic Acids Res. 49, D1138–D1143. doi: 10.1093/nar/gkaa891, PMID: 33068428 PMC7779006

[ref9] De Lazzari F. Bisaglia M. Zordan M. A. Sandrelli F. (2018). Circadian rhythm abnormalities in Parkinson's Disease from humans to flies and Back. Int. J. Mol. Sci. 19:3911. doi: 10.3390/ijms19123911 30563246 PMC6321023

[ref10] Earls R. H. Lee J. K. (2020). The role of natural killer cells in Parkinson's disease. Exp. Mol. Med. 52, 1517–1525. doi: 10.1038/s12276-020-00505-7, PMID: 32973221 PMC8080760

[ref11] Earls R. H. Menees K. B. Chung J. Gutekunst C. A. Lee H. J. Hazim M. G.. (2020). NK cells clear α-synuclein and the depletion of NK cells exacerbates synuclein pathology in a mouse model of α-synucleinopathy. Proc. Natl. Acad. Sci. USA 117, 1762–1771. doi: 10.1073/pnas.1909110117, PMID: 31900358 PMC6983411

[ref12] Emamzadeh F. N. Surguchov A. (2018). Parkinson's Disease: biomarkers, treatment, and risk factors. Front. Neurosci. 12:612. doi: 10.3389/fnins.2018.00612, PMID: 30214392 PMC6125353

[ref13] Franz M. Rodriguez H. Lopes C. Zuberi K. Montojo J. Bader G. D.. (2018). GeneMANIA update 2018. Nucleic Acids Res. 46, W60–W64. doi: 10.1093/nar/gky311, PMID: 29912392 PMC6030815

[ref14] Garaulet M. Ordovás J. M. Gómez-Abellán P. Martínez J. A. Madrid J. A. (2011). An approximation to the temporal order in endogenous circadian rhythms of genes implicated in human adipose tissue metabolism. J. Cell. Physiol. 226, 2075–2080. doi: 10.1002/jcp.22531, PMID: 21520059 PMC4428936

[ref15] Garcia-Esparcia P. Hernández-Ortega K. Ansoleaga B. Carmona M. Ferrer I. (2015). Purine metabolism gene deregulation inParkinson's disease. Neuropathol. Appl. Neurobiol. 41, 926–940. doi: 10.1111/nan.12221 25597950

[ref16] Garg A. Sinha S. (2022). Doxorubicin induced aggregation of α-synuclein: insights into the mechanism of drug induced parkinsonism. Colloids Surf. B Biointerfaces 212:112371. doi: 10.1016/j.colsurfb.2022.112371, PMID: 35131711

[ref17] Gros P. Videnovic A. (2020). Overview of sleep and circadian rhythm disorders in Parkinson Disease. Clin. Geriatr. Med. 36, 119–130. doi: 10.1016/j.cger.2019.09.005, PMID: 31733692 PMC6921931

[ref18] Grumati P. Morozzi G. Hölper S. Mari M. Harwardt M. I. Yan R.. (2017). Full length RTN3 regulates turnover of tubular endoplasmic reticulum via selective autophagy. eLife 6:e25555. doi: 10.7554/eLife.25555, PMID: 28617241 PMC5517149

[ref19] Guatteo E. Berretta N. Monda V. Ledonne A. Mercuri N. B. (2022). Pathophysiological features of Nigral dopaminergic neurons in animal models of Parkinson's Disease. Int. J. Mol. Sci. 23:4508. doi: 10.3390/ijms23094508, PMID: 35562898 PMC9102081

[ref20] Hood S. Amir S. (2017). The aging clock: circadian rhythms and later life. J. Clin. Invest. 127, 437–446. doi: 10.1172/JCI90328, PMID: 28145903 PMC5272178

[ref21] Hunt J. Coulson E. J. Rajnarayanan R. Oster H. Videnovic A. Rawashdeh O. (2022). Sleep and circadian rhythms in Parkinson's disease and preclinical models. Mol. Neurodegener. 17:2. doi: 10.1186/s13024-021-00504-w, PMID: 35000606 PMC8744293

[ref22] Jankovic J. Tan E. K. (2020). Parkinson's disease: etiopathogenesis and treatment. J. Neurol. Neurosurg. Psychiatry 91, 795–808. doi: 10.1136/jnnp-2019-322338 32576618

[ref23] Jia A. Yang Z. W. Shi J. Y. Liu J. M. Zhang K. Cui Y. F. (2022). MiR-325-3p alleviates acute pancreatitis via targeting RIPK3. Dig. Dis. Sci. 67, 4471–4483. doi: 10.1007/s10620-021-07322-6 35094251

[ref24] Keenan A. B. Torre D. Lachmann A. Leong A. K. Wojciechowicz M. L. Utti V.. (2019). ChEA3: transcription factor enrichment analysis by orthogonal omics integration. Nucleic Acids Res. 47, W212–W224. doi: 10.1093/nar/gkz446, PMID: 31114921 PMC6602523

[ref25] Kim S. Chen J. Cheng T. Gindulyte A. He J. He S.. (2023). PubChem 2023 update. Nucleic Acids Res. 51, D1373–D1380. doi: 10.1093/nar/gkac956, PMID: 36305812 PMC9825602

[ref26] Krämer A. Green J. Pollard J. Jr. Tugendreich S. (2014). Causal analysis approaches in ingenuity pathway analysis. Bioinformatics 30, 523–530. doi: 10.1093/bioinformatics/btt703 24336805 PMC3928520

[ref27] Kulisevsky J. Luquin M. R. Arbelo J. M. Burguera J. A. Carrillo F. Castro A.. (2013). Advanced Parkinson's disease: clinical characteristics and treatment (part 1). Neurologia 28, 503–521. doi: 10.1016/j.nrl.2013.05.001, PMID: 23856182

[ref28] Labib A. Y. Ammar R. M. El-Naga R. N. El-Bahy A. Tadros M. G. Michel H. E. (2021). Mechanistic insights into the protective effect of paracetamol against rotenone-induced Parkinson's disease in rats: possible role of endocannabinoid system modulation. Int. Immunopharmacol. 94:107431. doi: 10.1016/j.intimp.2021.107431, PMID: 33578261

[ref29] Langfelder P. Horvath S. (2008). WGCNA: an R package for weighted correlation network analysis. BMC Bioinformatics 9:559. doi: 10.1186/1471-2105-9-559, PMID: 19114008 PMC2631488

[ref30] Li J. H. Liu S. Zhou H. Qu L. H. Yang J. H. (2014). starBase v2.0: decoding miRNA-ceRNA, miRNA-ncRNA and protein-RNA interaction networks from large-scale CLIP-Seq data. Nucleic Acids Res. 42, D92–D97. doi: 10.1093/nar/gkt1248, PMID: 24297251 PMC3964941

[ref31] Li S. Shui K. Zhang Y. Lv Y. Deng W. Ullah S.. (2017). CGDB: a database of circadian genes in eukaryotes. Nucleic Acids Res. 45, D397–D403. doi: 10.1093/nar/gkw1028, PMID: 27789706 PMC5210527

[ref32] Liu T. T. Li R. Huo C. Li J. P. Yao J. Ji X. L.. (2021). Identification of CDK2-related immune forecast model and ceRNA in lung adenocarcinoma, a Pan-Cancer analysis. Front. Cell Dev. Biol. 9:682002. doi: 10.3389/fcell.2021.682002, PMID: 34409029 PMC8366777

[ref33] Maiti P. Manna J. Dunbar G. L. (2017). Current understanding of the molecular mechanisms in Parkinson's disease: targets for potential treatments. Transl Neurodegener 6:28. doi: 10.1186/s40035-017-0099-z, PMID: 29090092 PMC5655877

[ref34] McGeary S. E. Lin K. S. Shi C. Y. Pham T. M. Bisaria N. Kelley G. M.. (2019). The biochemical basis of microRNA targeting efficacy. Science 366:eaav1741. doi: 10.1126/science.aav1741, PMID: 31806698 PMC7051167

[ref35] Miki Y. Shimoyama S. Kon T. Ueno T. Hayakari R. Tanji K.. (2018). Alteration of autophagy-related proteins in peripheral blood mononuclear cells of patients with Parkinson's disease. Neurobiol. Aging 63, 33–43. doi: 10.1016/j.neurobiolaging.2017.11.006, PMID: 29223072

[ref36] Möller-Levet C. S. Archer S. N. Bucca G. Laing E. E. Slak A. Kabiljo R.. (2013). Effects of insufficient sleep on circadian rhythmicity and expression amplitude of the human blood transcriptome. Proc. Natl. Acad. Sci. USA 110, E1132–E1141. doi: 10.1073/pnas.1217154110, PMID: 23440187 PMC3607048

[ref37] Musachio E. Araujo S. M. Bortolotto V. C. de Freitas Couto S. Dahleh M. Poetini M. R.. (2020). Bisphenol a exposure is involved in the development of Parkinson like disease in Drosophila melanogaster. Food Chem. Toxicol. 137:111128. doi: 10.1016/j.fct.2020.111128, PMID: 31952986

[ref38] Mutez E. Larvor L. Leprêtre F. Mouroux V. Hamalek D. Kerckaert J. P.. (2011). Transcriptional profile of Parkinson blood mononuclear cells with LRRK2 mutation. Neurobiol. Aging 32, 1839–1848. doi: 10.1016/j.neurobiolaging.2009.10.016 20096956

[ref39] Ooi S. Jiang H. Kang Y. Allard P. (2021). Examining the developmental trajectory of an in vitro model of mouse primordial germ cells following exposure to environmentally relevant Bisphenol a levels. Environ. Health Perspect. 129:97013. doi: 10.1289/EHP8196, PMID: 34585602 PMC8480152

[ref40] Ptak A. Gregoraszczuk E. L. (2012). Bisphenol a induces leptin receptor expression, creating more binding sites for leptin, and activates the JAK/stat, MAPK/ERK and PI3K/Akt signalling pathways in human ovarian cancer cell. Toxicol. Lett. 210, 332–337. doi: 10.1016/j.toxlet.2012.02.003, PMID: 22343039

[ref41] Reiter R. J. Sharma R. Ma Q. (2021). Switching diseased cells from cytosolic aerobic glycolysis to mitochondrial oxidative phosphorylation: a metabolic rhythm regulated by melatonin. J. Pineal Res. 70:e12677. doi: 10.1111/jpi.12677, PMID: 32621295

[ref42] Robin X. Turck N. Hainard A. Tiberti N. Lisacek F. Sanchez J. C.. (2011). pROC: an open-source package for R and S+ to analyze and compare ROC curves. BMC Bioinform. 12:77. doi: 10.1186/1471-2105-12-77, PMID: 21414208 PMC3068975

[ref43] Serin Y. Acar Tek N. (2019). Effect of circadian rhythm on metabolic processes and the regulation of energy balance. Ann. Nutr. Metab. 74, 322–330. doi: 10.1159/000500071 31013492

[ref44] Sharoar M. G. Yan R. (2017). Effects of altered RTN3 expression on BACE1 activity and Alzheimer's neuritic plaques. Rev. Neurosci. 28, 145–154. doi: 10.1515/revneuro-2016-0054 27883331

[ref45] Shin J. H. Ko H. S. Kang H. Lee Y. Lee Y. I. Pletinkova O.. (2011). PARIS (ZNF746) repression of PGC-1α contributes to neurodegeneration in Parkinson's disease. Cell 144, 689–702. doi: 10.1016/j.cell.2011.02.010, PMID: 21376232 PMC3063894

[ref46] Shkodina A. D. Tan S. C. Hasan M. M. Abdelgawad M. Chopra H. Bilal M.. (2022). Roles of clock genes in the pathogenesis of Parkinson's disease. Ageing Res. Rev. 74:101554. doi: 10.1016/j.arr.2021.101554 34973458

[ref47] Spira J. L. Spiegel D. (1992). Hypnosis and related techniques in pain management. Hosp. J. 8, 89–119. doi: 10.1300/J011v08n01_05 1286854

[ref48] Stoker T. B. Greenland J. C. (2018). Parkinson’s Disease: Pathogenesis and clinical aspects. Brisbane (AU): Codon Publications. 30702835

[ref49] Su R. Jin C. Bu H. Xiang J. Zhou L. Jin C. (2022). Development and validation of an immune-related prognostic signature in cervical Cancer. Front. Oncol. 12:861392. doi: 10.3389/fonc.2022.861392, PMID: 35651784 PMC9148954

[ref50] Surguchov A. Surguchev A. (2022). Synucleins: new data on Misfolding, aggregation and role in diseases. Biomedicines 10:3241. doi: 10.3390/biomedicines10123241, PMID: 36551997 PMC9775291

[ref51] Thongkorn S. Kanlayaprasit S. Jindatip D. Tencomnao T. Hu V. W. Sarachana T. (2019). Sex differences in the effects of prenatal Bisphenol a exposure on genes associated with autism Spectrum disorder in the Hippocampus. Sci. Rep. 9:3038. doi: 10.1038/s41598-019-39386-w, PMID: 30816183 PMC6395584

[ref52] Tolosa E. Garrido A. Scholz S. W. Poewe W. (2021). Challenges in the diagnosis of Parkinson's disease. Lancet Neurol. 20, 385–397. doi: 10.1016/S1474-4422(21)00030-2, PMID: 33894193 PMC8185633

[ref53] Verheijen M. Schrooders Y. Gmuender H. Nudischer R. Clayton O. Hynes J.. (2018). Bringing in vitro analysis closer to in vivo: studying doxorubicin toxicity and associated mechanisms in 3D human microtissues with PBPK-based dose modelling. Toxicol. Lett. 294, 184–192. doi: 10.1016/j.toxlet.2018.05.029, PMID: 29803840

[ref54] Videnovic A. Zee P. C. (2015). Consequences of circadian disruption on neurologic health. Sleep Med. Clin. 10, 469–480. doi: 10.1016/j.jsmc.2015.08.004, PMID: 26568123 PMC4648713

[ref55] Vijay V. Moland C. L. Han T. Fuscoe J. C. Lee T. Herman E. H.. (2016). Early transcriptional changes in cardiac mitochondria during chronic doxorubicin exposure and mitigation by dexrazoxane in mice. Toxicol. Appl. Pharmacol. 295, 68–84. doi: 10.1016/j.taap.2016.02.003, PMID: 26873546

[ref56] Wang Y. Wang Z. Sun J. Qian Y. (2021). Identification of HCC subtypes with different prognosis and metabolic patterns based on Mitophagy. Front. Cell Dev. Biol. 9:799507. doi: 10.3389/fcell.2021.799507, PMID: 34977039 PMC8716756

[ref57] Wang H. Zhou Z. Liu Y. Wang P. Chen L. Qi S.. (2022). Identification and validation of HOXD3 and UNC5C as molecular signatures in keloid based on weighted gene co-expression network analysis. Genomics 114:110403. doi: 10.1016/j.ygeno.2022.110403, PMID: 35709926

[ref58] Yang W. Hamilton J. L. Kopil C. Beck J. C. Tanner C. M. Albin R. L.. (2020). Current and projected future economic burden of Parkinson's disease in the U.S. NPJ Parkinson's Dis. 6:15. doi: 10.1038/s41531-020-0117-1, PMID: 32665974 PMC7347582

[ref59] Yang Y. Wu X. Lu X. Wang C. Xiang L. Zhang C. (2022). Identification and validation of autophagy-related genes in vitiligo. Cells 11:1116. doi: 10.3390/cells11071116, PMID: 35406685 PMC8997611

[ref60] Yin Z. Deng J. Zhou M. Li M. Zhou E. Liu J.. (2022). Exploration of a novel circadian miRNA pair signature for predicting prognosis of lung adenocarcinoma. Cancers (Basel) 14:5106. doi: 10.3390/cancers14205106, PMID: 36291889 PMC9600995

[ref61] Yin X. Wang M. Wang W. Chen T. Song G. Niu Y.. (2022). Identification of potential miRNA-mRNA regulatory network contributing to Parkinson's Disease. Parkinson's Dis. 2022:2877728. doi: 10.1155/2022/2877728, PMID: 36105301 PMC9467752

[ref62] Yu G. Wang L. G. Han Y. He Q. Y. (2012). clusterProfiler: an R package for comparing biological themes among gene clusters. OMICS 16, 284–287. doi: 10.1089/omi.2011.0118, PMID: 22455463 PMC3339379

[ref63] Yu D. Wu L. Gill P. Tolleson W. H. Chen S. Sun J.. (2018). Multiple microRNAs function as self-protective modules in acetaminophen-induced hepatotoxicity in humans. Arch. Toxicol. 92, 845–858. doi: 10.1007/s00204-017-2090-y 29067470 PMC5820133

[ref64] Zhang T. M. Yu S. Y. Guo P. Du Y. Hu Y. Piao Y. S.. (2016). Nonmotor symptoms in patients with Parkinson disease: a cross-sectional observational study. Medicine (Abingdon) 95:e5400. doi: 10.1097/MD.0000000000005400, PMID: 27977578 PMC5268024

